# Health-related quality of life and symptom burden in patients with melanoma during and after immune checkpoint inhibitor therapy – a pilot study

**DOI:** 10.1186/s12885-025-15069-w

**Published:** 2025-10-16

**Authors:** Lea Pöschmann, Friedegund Meier, Martin Eichler, Frank Friedrich Gellrich, Jochen Schmitt, Olaf Schoffer

**Affiliations:** 1 Department of Dermatology, Faculty of Medicine and University Hospital Carl Gustav Carus, TUD Dresden University of Technology, Fetscherstraße 74, 01307 Dresden, Germany; 2 Skin Cancer Center at the National Center for Tumor Diseases and University Cancer Centre Dresden, Fetscherstraße 74, Dresden, 01307 Germany; 3 National Center for Tumor Diseases (NCT/UCC), Faculty of Medicine and University Hospital Carl Gustav Carus, TUD Dresden University of Technology, Fetscherstraße 74, 01307 Dresden, Germany; 4 Center for Evidence-Based Healthcare, Faculty of Medicine and University Hospital Carl Gustav Carus, TUD Dresden University of Technology, Fetscherstraße 74, 01307 Dresden, Germany

**Keywords:** Immune checkpoint inhibitor, Immunotherapy, Melanoma, HRQoL, Survivorship

## Abstract

**Background:**

Measurement of health-related quality of life (HRQoL) in patients with melanoma under immune checkpoint inhibitor therapy (ICI) under routine conditions and in survivorship is insufficient due to the absence of data and therapy-specific instruments in clinical trials. This pilot study aimed to collect and compare data on symptom burden and HRQoL in patients during and after ICI therapy. The benefit of a treatment-/disease-specific item list compared to a general-oncologic HRQoL instrument was examined. The results are intended to be used for planning a longitudinal study.

**Methods:**

This cross-sectional, monocentric, questionnaire-based study included patients with melanoma stages II-IV treated at the University Hospital Dresden, Germany. HRQoL was measured in a treatment group (ongoing ICI therapy) and a survivor group (minimum one year therapy- and relapse-free after ICI). The treatment group was interviewed with the cancer-generic EORTC QLQ-C30 and the survivor group with the EORTC SURV100, both consisting of different scales (range 0-100). Hereby, an elevated HRQoL score indicates a better HRQoL. Additionally, a treatment-/disease-specific PRO-CTCAE item list was conducted in both groups. We performed descriptive and exploratory analysis aiming to identify and compare symptom burden in both groups.

**Results:**

General health measured with EORTC C30/SURV100 in the treatment group (73 patients) was marginally lower than in the survivor group (26 patients) with a mean score of 61.0 (SD ± 21) vs. 67.0 (SD ± 21), which implies descriptively lower HRQoL scores in the treatment group than in clinical trials and population norm data. With almost similar demographic-clinical characteristics, the treatment group frequently reported pruritus and xerostomia, while survivors frequently reported aching joints, memory loss and a considerable emotional burden. The high prevalence of xerosis cutis, fatigue and sexual dysfunction was observed across both groups. In both groups, the specific PRO-CTCAE identified further relevant symptoms than the general instruments.

**Conclusions:**

Needs of patients during and after ICI therapy appear to differ with an assumed remaining symptom burden in survivors of melanoma. For measuring HRQoL a therapy-/disease-specific instrument should be considered. Rising numbers of patients undergoing ICI therapy and survivors underscore the need for comprehensive data on HRQoL and specific aspects of treatment.

**Supplementary Information:**

The online version contains supplementary material available at 10.1186/s12885-025-15069-w.

## Background

Melanoma, also known as malignant melanoma, is an aggressive tumour of the skin [1]. Since the introduction of immune checkpoint inhibitor (ICI) therapy for the advanced stages, there was an important change in treatment. The mechanism of action of ICI involves the modulation of T-cell, antigen-presenting cell and tumour cell interactions, thereby enhancing the response of the immune system against the tumour [2]. However, ICI-induced immune-related adverse events (irAEs) remain a clinical challenge because they are very common and may be severe, irreversible and even lethal [3]. Clinical trials showed high toxicity, particularly for the combination therapy with ipilimumab and nivolumab with a high rate (59%) of severe irAEs [4].

Nevertheless, immune checkpoint inhibitors significantly improved overall survival [5]. This observation led to a growing interest in survivorship as subject of research [6]. There is no clear definition of the term “survivor”, but it describes the transition from diagnosis and treatment to long-term survival with specific challenges, including long-term toxicities [7].

Another quality criterion in oncology with increasing relevance, alongside survival, is health-related quality of life (HRQoL) [8]. This multi-dimensional construct includes different aspects, such as physical, social and psychological function [9]. The use of patient-reported outcomes (PRO) for measuring HRQoL has gained in importance. Frequently used instruments are questionnaires in which patients self-report their HRQoL [10, 11].

However, both data on HRQoL under routine conditions in patients with melanoma under ICI therapy and data on survivors of melanoma after ICI therapy have been insufficient so far and outcomes of clinical trials and real-world data may differ [12]. Clinical trials showed that the HRQoL of patients receiving ICI did not decrease more compared to the control group despite high toxicity of ICI [13, 14]. Comparing the HRQoL under ICI therapy with HRQoL under chemotherapy the results of the KEYNOTE 002 trial showed that patients undergoing pembrolizumab therapy experienced significantly better HRQoL than patients undergoing chemotherapy [15]. The problem is that clinical trials frequently used unspecific general-oncologic HRQoL instruments which may not measure all symptoms pertinent to patients with ICI therapy [16]. To our knowledge, no real-world study has yet compared HRQoL in patients during ICI treatment and in long-term survivors after ICI therapy. Therefore, the long-term course of HRQoL after the end of therapy remains unclear. Furthermore, it is unclear how long irAEs affect HRQoL, or how strong the impact of the therapy success or failure is, independently of irAEs. Additionally, survivors may have different needs than patients with ongoing therapy [7]. This knowledge is necessary to better understand the specific needs of patients and to guarantee an individualised follow-up including HRQoL assessment and measurement. Thus, the aim of this pilot study was to measure and compare data on symptom burden and HRQoL under routine conditions of patients with melanoma under ICI therapy (treatment group) with patients with melanoma after ICI therapy (survivor group) to identify relevant symptoms and differences that may affect follow-up, paying particular attention to specific symptoms. It was examined whether the discrepancy in HRQoL and toxicity observed in clinical trials could be attributed to the measurement of HRQoL under routine conditions. The study also examined the HRQoL using a disease- and therapy-specific instrument.

The results of our study could serve as a basis for a multicentric, longitudinal trial focused on HRQoL. In the context of the exploratory character of this study, new hypotheses should be formulated during the course of the study.

## Methods

### Population sample and study design

We performed a monocentric, cross-sectional study with exploratory character including patients from the skin cancer department of the university hospital in Dresden, Germany.

The study was conducted with the approval of the ethics committee of the Technical University Dresden (reference number: SR + BO-EK-184042023). Patients were enrolled between June 1, 2023 to December 31, 2023. Patients with histologically confirmed melanoma stages II-IV (American Joint Committee on Cancer [AJCC] version 8) aged at least 18 years were included in the study. For the treatment group, recent ICI therapy either as monotherapy with pembrolizumab or nivolumab, or as combination therapy with ipilimumab and nivolumab was required. Patients in the survivor group were required to be free of recurrence since their last ICI infusion and to have been off-treatment for a minimum of one year. General inclusion criteria encompassed the ability to complete questionnaires what includes speaking German, no cognitive diseases and providing an informed consent. There were no exclusion criteria for comorbidities or uveal or mucosal melanoma. Patients in the survivor group who had experienced recurrence or any subsequent treatment for melanoma after ICI treatment were excluded from the study.

Eligible patients of both groups were interviewed during their appointments in the university hospital. The participants were thoroughly apprised of the procedure and following their consent for participation, the questionnaires were administered. To minimise the non-response rate the patients were informed very detailed about the study aims. Patients in the survivor group who did not have an appointment scheduled at the hospital, were contacted postally. The same person (unknown to the patients) mainly provided information about the study and handed out the study documents to minimise the response bias. The interviews were conducted once, at a single point in time. Due to the exploratory study design, a calculation of the number of cases required for recruitment wasn’t conducted. Instead, we did an assessment on the expected number of cases, which was deemed realistic for a recruitment period of seven months. It was anticipated that approximately 60 patients would be enrolled in the treatment group, while the survivor group was expected to comprise around 50 patients. The number of patients expected in the survivor group was expected to be lower due to the occurrence of relapses or additional therapy after ICI in multiple patients. The study is reported according to the STROBE statement.

### Data collection

The clinical-demographic data encompassed various patient-related characteristics, including age, gender, tumour stage, therapy regime, previous therapies, comorbidities, performance status and adverse events. These data were collected using the electronic documentation system of the hospital. Data on HRQoL were self-reported as patient-reported outcomes by the patients (questionnaires carried out on paper). The primary objectives were to assess and compare HRQoL and symptom burden in both groups. As second objective we evaluated descriptively whether a therapy-specific instrument could improve HRQoL measurement.

### HRQoL instruments

Study participants were required to complete two questionnaires: a general-oncologic questionnaire and additionally a disease-/treatment-specific item list. The treatment group was required to complete the EORTC QLQ-C30 version 3.0 and the survivor group the EORTC SURV100 (recent version after phase 3 testing) as general-oncologic instruments [17, 18]. Both groups were asked to complete the specific PRO-CTCAE item list [19]. The instruments utilised are outlined in Table 1. The patients were asked to report any ongoing symptoms at the time of the survey.

**Table 1:** Used HRQoL instruments

Treatment group		Survivor group
EORTC QLQ-C30	general-oncologic instrument	EORTC SURV100
PRO-CTCAE	disease-/therapy-specific instrument	PRO-CTCAE

The EORTC QLQ-C30 is a well-validated and reliable questionnaire for patients with cancer and is frequently utilised in clinical trials [17, 20]. It comprises symptom scales, functional scales and a global health score [17]. The EORTC SURV100 was developed for cancer survivors and consists of items with a 4-point Likert scale, only health score and quality of life could be rated on a 7-point Likert scale [18]. The items of both questionnaires correspond to symptom and functional scales, ranging from 0 to 100 [17, 18]. An elevated score on the symptom scales indicates a higher symptom burden. In contrast, an elevated score on the functional scales and the quality-of-life scale denotes an enhanced function/quality of life [21]. The SURV100 additionally consists of scales concerning survivor issues, such as fear of recurrence or personal growth [18].

The PRO-CTCAE items were developed by the National Cancer Institute with the objective of constructing specific questionnaires for diverse patient populations [19]. The items of our PRO-CTCAE item list were selected by expert opinion. The inclusion of items was predicated on their anticipated relevance as adverse events in patients undergoing ICI therapy. The final questionnaire comprised 24 items, in addition to an open-ended question for the reporting of other symptoms. The items could be rated by a 5-point Likert scale and contained different attributes like frequency, strength and interference (impact on daily activities) of the symptom [19].

Questionnaires that contained one or more missing items in the PRO-CTCAE or less than 50% of the items for one scale in the EORTC questionnaires were excluded [21]. According to literature there are different thresholds for a minimally important difference. In clinical trials assessing HRQoL in patients receiving ICI therapy, a difference of 10 points is usually considered relevant [15]. Overlapping symptoms were not cross-validated between the self-reports and the data derived from electronic health records. However, we performed a validation of similar items in the EORTC and PRO-CTCAE questionnaire It is known that self-reported symptoms and physician assessment differ but comparing this was not one of our aims [22]. Additionally, the outcomes of the data sources should not be changed.

### Statistical analysis

Demographic-clinical characteristics were analysed by descriptive statistics which included mean, standard deviation, absolute and relative frequencies. The results of the EORTC questionnaires were calculated in accordance with the scoring manual and were described with mean, range and standard deviation [21]. The results of the PRO-CTCAE were described with relative and absolute frequencies, because there is no general scoring procedure.

In order to facilitate a meaningful comparison of the results of the EORTC QLQ-C30 between the treatment and survivor group, we calculated p-values for every scale of the EORTC QLQ-C30 with Mann-Whitney-U-Test. For this analysis, the values of the C30 scales for the survivor group were calculated using the SURV100. The C30 scales were calculated using the original items of the C30. P-values of ≤0.05 were considered as relevant. Due to the pilot status of the study, all test results, including p-values, should be interpreted as exploratory. To facilitate exploratory analysis for the scales of the EORTC QLQ-C30 of the treatment group, a subgroup analysis was performed for sex (male vs. female), treatment agent (nivolumab vs. pembrolizumab vs. ipilimumab/nivolumab), age (< 50 years vs. 50–59 years vs. 60–69 years vs. ≥70 years), ECOG (0 vs. 1 vs. 2–3), brain metastases (yes vs. no) and irAEs (yes vs. no). For this, we used the Mann-Whitney-U-Test (for two subgroups) and Kruskal-Wallis Test (for three or more subgroups) testing statistical differences between the subgroups. For the Kruskal-Wallis Tests a post hoc analysis was conducted using the Dunn test to evaluate which subgroups differ significantly. These analyses should identify possible clinical characteristics for a better or lower HRQoL or particular symptoms.

Analyses and hypotheses were pre-specified due to the study protocol (see supplementary material). Independently, the exploratory character also was pre-specified. All analyses were performed using R version 2023.06.1 + 524 with the packages correlation, corrplot, psych, car and rstatix.

## Results

**Fig. 1:**
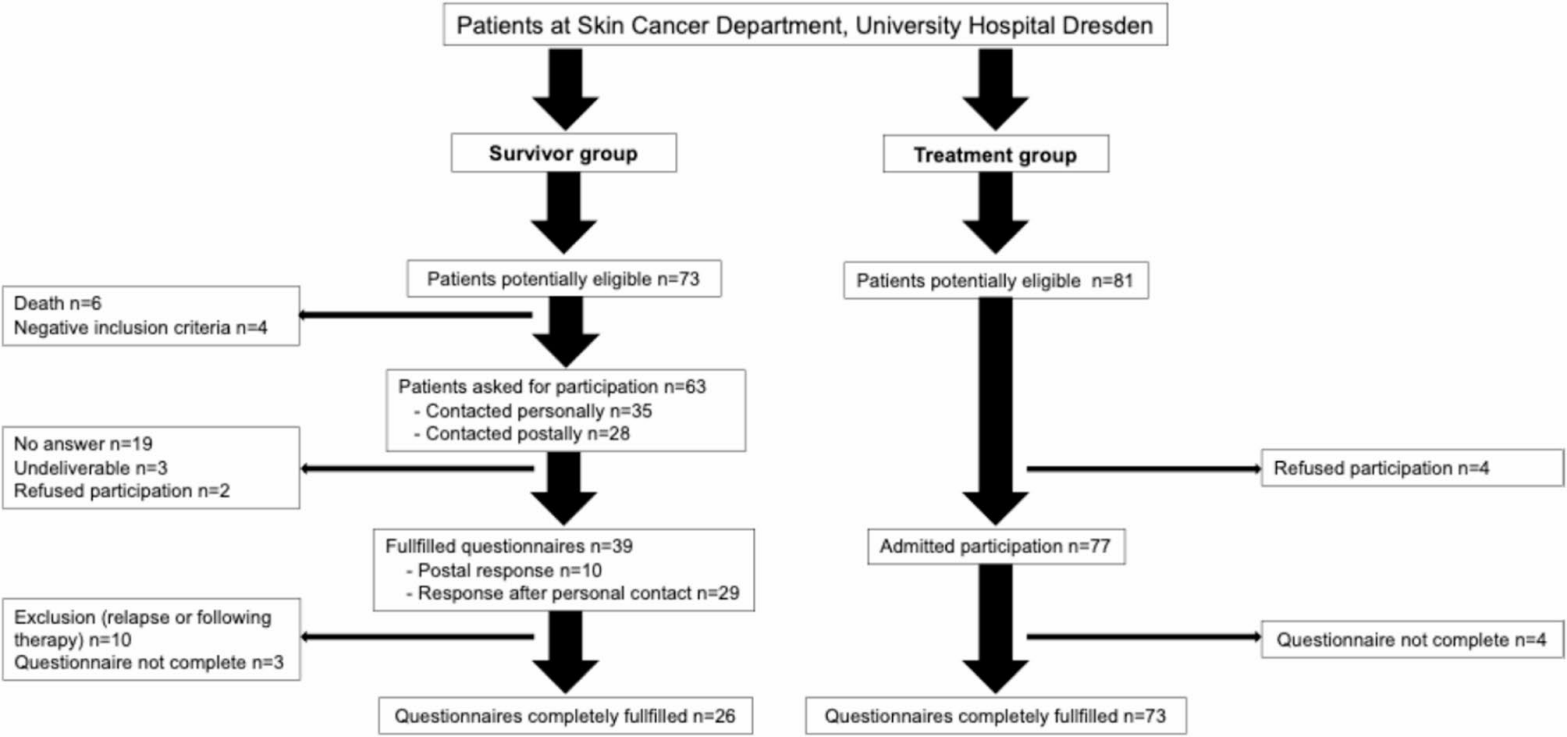
Patient selection flowchart. *N* number of patients

In the treatment group 81 patients were asked for participation. Four patients declined their participation, so 77 patients received the questionnaires. Four patients were excluded because of incomplete questionnaires, resulting in a final sample of 73 patients for the treatment group (response rate 90%). For the survivor group a request in the institutional database and the internal tumour documentation system of the University Hospital Dresden identified 73 potentially eligible patients. Six were excluded because of death. The participants were approached in two ways: 35 were asked for participation during their appointment in the hospital, 28 patients were contacted postally. The response rate was 41%. The response rate for personal contact was higher than for postal contact. Out of the remaining 39 patients, 10 patients had to be excluded because they relapsed and another 3 because of missing items in the questionnaires, so we finally included 26 patients in the survivor group. The patient selection process is illustrated in Fig. 1.

### Demographic-clinical characteristics

For the treatment group the mean age was 67.5 years (± 14.5 SD) (see Table 2). 45% of patients were female and 55% male. Patients were mainly in advanced melanoma stage (according to American Joint Committee of Cancer, version 8: stage IV 64.4%, stage III 21.8%, stage II 9.7%), for the remaining three patients the tumour stage was unclear between III and IV. 23% had confirmed brain metastases. 20% in the treatment group received combined therapy, 55% nivolumab and 23% pembrolizumab. 59% received ICI as palliative treatment. Most patients (64%) were maximum three months on treatment. 18% were hospitalised at time of survey. The majority of patients had received prior treatment and showed multiple comorbidities.

In the survivor group the mean age was 69.5 years (± 15.1 SD). 34.6% of the patients was female. Tumour stage was about predominantly advanced (3.8% stage II, 38.5% stage III and 57.7% stage IV). Brain metastases were detected in 15% of the patients. One patient had received combined therapy, the percentages for pembrolizumab and nivolumab were almost similar. For 51% of the survivors the former ICI therapy was indicated as palliative treatment. 77% had responded to ICI treatment and 23% had discontinued treatment due to toxicity. Most patients were in year 2 to 4 after treatment. Multiple patients were pretreated and had several comorbidities.

**Table 2:** Clinical-demographic characteristics of the treatment and survivor group

Characteristics	Treatment group (*n* = 73)	Survivor group (*n* = 26)
Gender – *n* (%)		
male	40 (54.8)	17 (65.4)
female	33 (45.2)	9 (34.6)
Age		
mean (SD)	67.5 years (± 14.5)	69.5 years (± 15.1)
range	18–92 years	33–88 years
Indication of therapy– n (%)		
adjuvant	30 (41.1)	12 (46.2)
palliative	43 (58.9)	14 (53.8)
Tumour stage (AJCC v8) – n (%)		
II	7 (9.7)	1 (3.8)
III	16 (21.9)	10 (38.5)
IV	47 (64.4)	15 (57.7)
unclear	3 (4.1)	0
Checkpointinhibitor therapy– n (%)		
Nivolumab	40 (54.8)	13 (50.0)
Pembrolizumab	17 (23.2)	12 (46.2)
Ipilimumab/Nivolumab	16 (21.9)	1 (3.8)
Brain metastases – n (%)	17 (23.3)	4 (15.4)
Setting – n (%)		
outpatient	60 (82.2)	26 (100)
inpatient	13 (17.8)	0
Development after ICI – n (%)		
CR/PR	Not applicable	20 (76.9)
discontinuation irAEs		6 (23.1)
Months undergoing ICI – n (%)		
≤6 months	54 (74.0))	Not collected
> 6 ≤12months	11 (15.1)	
> 12 ≤24 months	6 (8.3)	
> 24 months	2 (2.7)	
Months since end of ICI – n (%)		
12–23 months	Not applicable	4 (15.4)
24–35 months		5 (19.2)
36–47 months		11 (42.3)
≥ 48 months		7 (26.9)
ECOG at survey– n (%)		
0	37 (50.7)	16 (61.5)
1	26 (35.6)	9 (34.6)
2	9 (12.3)	0
3	1 (1.4)	1 (3.8)
Previous therapies – n (%)		
BRAF-/MEK-Inhibitor	3 (4.1)	2 (7.7)
further ICI	29 (39.7)	6 (23.1)
other systemic treatment	9 (12.3)	2 (7.7)
radiotherapy	21 (28.8)	14 (53.8)
no	40 (54.8)	10 (38.5)
Comorbidities – n (%)		
no	7 (9.6)	2 (7.7)
cardiovascular	45 (61.6)	15 (57.7)
neurologic	12 (16.4)	5 (19.2)
orthopedic	10 (13.7)	6 (23.1)
psychiatric	4 (5.5)	1 (3.8)
oncologic	13 (17.8)	7 (27.0)
pulmologic	5 (6.8)	0
endocrine	13 (17.8)	7 (27.0)
renal	9 (12.3)	5 (19.2)

*N* number, correlating percentages in brackets. Data based on electronic health records

### Immune-related adverse events

In the treatment group, 43% of patients had clinically documented (based on electronic health records) immune-related adverse events which were graded by CTCAE (see table S1). Colitis (11%), arthralgia (8,2%), hepatitis (8,2%) were most common. The combination therapy showed a higher number of severe adverse events (CTCAE grade 3–4) than nivolumab monotherapy (43.8% vs. 7.5%). For patients receiving pembrolizumab no severe irAEs were reported. Altogether, hepatitis (8.2%) and colitis (11%) occurred most frequently as severe.

46% of Survivors had documented irAEs (based on electronic health records) in their former checkpoint inhibitor treatment (see table S2, includes irAEs that occurred during and after the last ICI therapy), common were colitis (19%) and arthralgia (19,2%). CTCAE grading was not documented for this group.

### HRQoL and symptom burden of the treatment group

The mean HRQoL score in the treatment group was 61.0 (± 21.6 SD), as measured by the Global Health scale of the EORTC QLQ-C30 (shown in Table 3). Weighted Global Health by sex and age was 65.3. There was a good function in general, however, emotional function exhibited a marginal decline in comparison to the other functional scales. Highest scores in symptom scales were observed for fatigue with 39.4 (± 27.8) and insomnia with 30.6 (± 35). Scores for nausea/vomiting and constipation were the lowest A high prevalence of appetite loss was observed in the treatment group.

**Table 3:** Results of the EORTC QLQ-C30 in the treatment group

Scale	*n*	Mean	SD	Range
Global Health Score	73	61.0	21.6	0–100
Physical function	73	76.5	22.8	6.7–100
Role function	73	72.8	28.9	0–100
Emotional function	73	68.6	22.8	0–100
Cognitive function	73	85.2	20.8	16.7–100
Social function	73	75.8	26.2	0–100
Fatigue	73	39.4	27.8	0–100
Nausea/Vomiting	73	6.6	16.8	0–100
Pain	73	24.6	30.2	0–100
Dyspnoea	73	19.2	27.2	0–100
Insomnia	73	30.6	35.0	0–100
Appetite loss	73	19.2	29.4	0–100
Constipation	73	8.7	19.3	0–100
Diarrhea	73	10.0	24.3	0–100
Financial difficulties	73	10.0	20.6	0–100

*N* number pf patients, SD standard deviation

The most prevalent symptoms reported in the PRO-CTCAE in the treatment group were xerosis cutis (67.1%), pruritus (50.7%) and xerostomia (49.3%) (see Table 4, complete results shown in table S3). Approximately 50% of sexually active patients reported difficulties. Around 50% of men reported problems in achieving or sustaining an erection.

**Table 4:** Most prevalent symptoms in the PRO-CTCAE in the treatment group

Symptom	*n*	Absolute frequency	Relative frequency (%)
Xerosis cutis	73	49	67.1
Pruritus	73	37	50.7
Xerostomia	73	36	49.3
Problems erection	23	11	47.8
Reduced sexual interest	36	17	47.2
Aching joints	73	33	45.2
Memory loss	73	31	42.5
Hyp-/dysesthesia	73	30	41.1

Table contains reported symptoms with frequency > 40%. *N* number of patients

For the complete table with all symptoms see table S3 in the supplements

Symptoms that were reported mostly as often/almost constantly (means frequency CTCAE grades 3–4) were edema, aching joints and problems with ejaculation (see table S4). Xerosis cutis, pruritus, xerostomia and aching joints occurred mostly the strongest (means strength CTCAE grade 3–4) (see table S5). Edema, dysesthesia, and dizziness interfered most strongly with daily activities (means interference CTCAE grade 3 and 4) (see table S6).

Subsequent subgroup analysis of the QLQ-C30 in the treatment group by gender, checkpoint inhibitor and irAEs demonstrated differences in these subgroups. The analysis revealed that female patients showed lower HRQoL and predominantly higher symptom burden than men, particularly with regard to insomnia. Furthermore, patients undergoing combined therapy exhibited a diminished HRQoL in comparison to those administered monotherapy. Patients receiving ipilimumab/nivolumab reported more often fatigue, nausea, appetite loss and diarrhoea. Furthermore, patients who experienced irAEs exhibited a lower HRQoL and emotional function in comparison to those not experiencing such adverse effects. Data for subgroup analysis are shown in tables S7-S14.

### HRQoL and symptom burden of the survivor group

Survivors demonstrated a quality of life of 67.0 (± 19.4), shown in Table 5. Scales concerning future, change of behavior and symptom awareness were notably low, indicating a diminished functionality in comparison with the other functional scales (see Table 5, complete results shown in table S15). In symptom scales survivors showed high fatigue 34.3 (± 26.9), health distress 52.1 (± 32.2) and negative health outlook 38.6 (± 26.3). Concerns regarding the impact of the disease on family members and fear about recurrence were prevalent. Frequently reported symptoms in the SURV100 were aching joints, skin disorders, and problems in standing for a longer time. Sexually active reported a higher prevalence of sexual dysfunction.

**Table 5:** Selected scales of the EORTC SURV100

Scale	*n*	Mean	SD	Range
Functional scales				
Global Health Score	26	67.0	19.4	25.0–100.0
Physical function	26	69.0	26.8	0–100
Cognitive function	26	81.7	20.6	41.7–100
Emotional function	26	79.1	20.1	33.3–100
Role function	26	71.8	28.8	0–100
Symptom awareness	26	19.2	25.3	0–100
Positive health behavior change	26	34.6	34.0	0–100
Positive life outlook	26	44.9	31.1	0–100
Symptom scale				
Fatigue	26	34.3	26.9	0–83.3.3
Sleep	26	34.6	26.9	0–91.7.7
Health distress	26	52.1	32.2	0–100
Negative health outlook	26	38.6	26.3	4.8–90.5
Sexual problems	19	21.7	31.1	0–100
Single Items				
Risk of family members getting cancer	26	44.9	38.8	0–100
Deeper meaning to cancer	26	50.0	44.5	0–100
Worry impact cancer on child	22	68.2	33.3	0–100
Symptom checklist				
Aching joints	26	39.7	26.7	0–66.7.7
Skin problems	26	33.3	36.5	0–100
Difficulties for long standing	26	39.7	36.5	0–100

*N* number of responses, SD standard deviation

For the complete table with all scales see S15 in the supplements

In the PRO-CTCAE survivors mostly reported aching joints (76.9%), xerosis cutis (69.2%), dysesthesia (53.8%) and memory loss (53.8%) (see Table 7, full table S17 available in the supplements). Symptoms which occurred often/almost constantly were aching joints (23.1%) and edema (15.4%) (see table S4). Symptoms occurring mostly strong were aching joints (19.2%), xerosis cutis (15.4%) and difficulties in achieving or maintaining an erection among sexually active males (16.7%) (see table S5). Memory loss, cough and aching joints (all 7.7%), interfered the strongest with activities of daily life (see table S6). Reported symptoms were generally mild to moderate.

### Comparison treatment and survivor group

The clinical-demographic characteristics of the two groups were found to be quite similar. As important difference the used checkpoint inhibitors should be considered, in the treatment group 22% received combined therapy and in the survivor group only one patient.

Outcomes of the general oncologic questionnaires demonstrated slight differences, the only statistic with a p-value less than 0.05 was that of appetite loss (see Table 6, full table S16 available in the supplements including a comparison with German population norm data of the C30).

**Table 6:** Selected results of QLQ-C30 for both groups

Scale	Treatment group	Survivor group	*p*-value	MID
Global Health Score	61.0 (± 21.6)	67.0 (± 19.3)	0.207	
Physical function	76.5 (± 22.8)	76.7 (± 24.5)	0.898	
Role function	72.8 (± 28.9)	74.4 (± 28.8)	0.804	
Emotional function	68.6 (± 22.8)	74.0 (± 23.5)	0.311	
Cognitive function	85.2 (± 20.8)	82.1 (± 20.5)	0.393	
Social function	75.8 (± 26.2)	79.5 (± 19.6)	0.780	
Fatigue	39.4 (± 27.8)	35.5 (± 28.1)	0.550	
Pain	24.6 (± 30.2)	25.6 (± 21.7)	0.423	
Insomnia	30.6 (± 35.0)	35.9 (± 37.6)	0.539	
Appetite loss	19.2 (± 29.4)	6.7 (± 15.1)	0.042 *	*

For the survivor group scales were calculated with the help of SURV100

A *p*-value of < 0,05 was considered as significant

*MID* minimally important difference analogue to clinical trials, * means difference ³10 points

For the complete table with all scales see table S16 in the supplements

In the PRO-CTCAE we found different percentages of the symptoms between both groups which means potential differences in symptom burden. Frequencies of symptoms were mostly high (see Table 7, full table S17 available in the supplements).

**Table 7:** Selected results of PRO-CTCAE for both groups

Symptom	Treatment group			Survivor group		
	*n*	Absolut frequency	Relative frequency	*n*	Absolut frequency	Relative frequency
Xerosis cutis	73	49	67.1	26	18	69.2
Pruritus	73	37	50.7	26	12	46.2
Xerostomia	73	36	49.3	26	9	34.6
Problems erection	23	11	47.8	12	4	33.3
Reduced sexual interest	36	17	47.2	14	7	50.0
Aching joints	73	33	45.2	26	20	76.9
Memory loss	73	31	42.5	26	14	53.8
Hyp-/Dysesthesia	73	30	41.1	26	14	53.8

*N* number of patients

For the complete table with all items see table S17 in the supplements

## Discussion

Treatment of patients with melanoma with checkpoint inhibitor therapy remains challenging due to high toxicity. In our study HRQoL in the treatment group was descriptively marginally lower than in the survivor group. The treatment group frequently reported appetite loss and xerostomia, while survivors frequently reported aching joints, memory loss and a considerable emotional burden. Both groups showed frequently xerosis cutis, pruritus, fatigue and sexual dysfunction. In both groups, the specific PRO-CTCAE identified further relevant symptoms than the general instruments.

### Treatment group

In the present study, HRQoL of the treatment group (unweighted data) was slightly lower than German general population norm data although weighted HRQoL (by sex and age) was slightly higher [23]. However, it is important to consider the differences in patient characteristics, such as age and comorbidities, which may have contributed to the slight discrepancy, as reported. Comparing our results with other systemic therapies, for e.g. dabrafenib/trametinib, results of combi-d trial showed a Global Health Score in the QLQ-C30 of 70.7 at baseline for the dabrafenib/trametinib group which were maintained under therapy [24]. In the coBRIM trial in patients treated with vemurafenib/cobinimetinib HRQoL measured with the QLQ-C30 was 66.8 at baseline with no clinical meaningful changes under treatment [25]. These reported HRQoL scores were quite similar to our study. This indicates that both, BRAF-/MEK inhibition and immunotherapy maintain HRQoL [14, 24, 25].

Compared with chemotherapy, in the KEYNOTE 002 after 12 weeks of treatment patients with chemotherapy had a significant deterioration of the HRQoL and higher symptom burden compared to pembrolizumab [26]. Hereby, chemotherapy is less effective and more toxic than ICI resulting in a reduced HRQoL.

Looking at the different scales of the QLQ-C30 in the treatment group particularly role and emotional function were lower than population norm data [23]. Social impact on patients with melanoma has been previously reported, e.g. impact on social relationships [27]. Real-world data for adjuvant ICI in melanoma stages III/IV showed a stable HRQoL but some patients had deteriorated, social and emotional function at 12 months, especially younger patients what is consistent with our findings [28].

The treatment group showed high scores of fatigue, a well-documented side effect in clinical trials but fatigue is also high in general population [4, 23]. A further noteworthy observation is the prevalence of xerostomia, a novel finding that emerged under ICI therapy. While the extant data are scant, there are some publications describing checkpoint inhibitor-induced sicca syndrome [29].

In subgroup analysis minimally important differences or statistical anomalies were shown for gender, immune checkpoint inhibitor, stage, age and irAEs. The interpretation of the subgroup analysis is limited because of the small sample size. Male gender, younger age, early tumour stage and no irAEs were expected to have a positive impact on HRQoL [23]. The collected data suggest gender-specific differences regarding ICI treatment, what was already observed in patients with cancer [30, 31]. Comparing subgroup analysis data on checkpoint inhibition with clinical study data such as CheckMate 067 or Keynote-006, a lower HRQoL was observed in our treatment group [13, 14]. Baseline global health in CheckMate067 was 70.7 ± 22.3 for nivolumab combined with ipilimumab and 74.7 ± 19.4 for nivolumab monotherapy with no clinically meaningful changes during treatment [14]. Furthermore, our subgroup analysis for patients with and without irAE showed a clinically important difference in HRQoL in contrast to clinical trials. Indeed, there is a discussion ongoing to distinguish between outcomes of clinical trials and data in routine care [12]. Patients must fulfill certain criteria for inclusion in clinical trials, e.g. a good performance status. Additionally, HRQoL instruments were used that may not be suitable for patients receiving ICI therapy. Altogether, HRQoL outcomes may be better in clinical trials than in routine care.

### Survivor group

For our survivor group, HRQoL was equivalent to the population norm data [23]. Arthralgia was already reported in the study of Mamoor et al. with a similar percentage like in our study [32] Dysesthesia as a common symptom in our survivor group and as a possible neurological side effect of ICI is rare but often becomes chronic [33]. Comorbidities are also likely to have an impact. It is noteworthy that the psychological symptom burden in our survivor group is higher than the physical symptom burden. Fear of recurrence and anxiety is very common. A German study from showed the need for psychological support in a third of patients with melanoma [34]. This underscores psycho-oncological support during survivorship. In general, our results appear similar to existing real-world studies [32]. A real-world study from Belgium/Luxembourg confirmed stable under therapy. There, baseline HRQoL was 73.8 and but showing a deteriorated cognitive, emotional and social impairment that was higher after treatment than under [35]. This is consistent with our findings of the high emotional burden in survivors.

### Comparison of the treatment and survivor group

The disparity in HRQoL between our two groups was small and did not reach statistical significance. This must be considered in light of the small sample size and resulting low statistical power. The more frequent appetite loss in patients under ICI therapy than in the survivors could be a consequence of colitis as common side effect of ICI treatment [3]. This finding is the most frequently documented irAE in the treatment group, which may serve as a potential explanation for the observed differences. Survivors reported more frequently memory loss than the treatment group. Impacts of ICI on neurocognition still remain unclear [36].

Sexual dysfunction was prevalent in both groups. Effects of a treatment with an immune checkpoint inhibitor on fertility still remain unclear but estimates for Germany in general suggest sexual dysfunction in 33% of men ad 46% of women [37–39].

These data suggest that there are different symptoms in patients during and after ICI therapy. Comparing the needs and symptoms of patients during and after therapy, it is important to know the impact of side effects on HRQoL and how long this may remain. Furthermore, HRQoL is next to tumour response an important fact for the success of the treatment. The different needs of the patients show that there should be a specific approach for every patient population due to the standing in the cancer therapy including different HRQoL instruments. Possible remaining gaps in our routine care and follow-up have to be identified and improved to guarantee an appropriate patient care.

### HRQoL instruments

A difference in symptom burden between the survivor and treatment group was visible, especially in the PRO-CTCAE (only descriptive comparison). Different symptoms between the groups were mainly xerostomia, aching joints, memory loss or dysesthesia. Looking at the two general-oncologic questionnaires there were hardly any differences between the groups. General scales in the general-oncologic questionnaires, such as global health or emotional function are intended for any patient population. Our study showed that survivors do not really have better function regarding these general scales. This may be related to long-term toxicities or psychological distress. All questionnaires contained items that were reported by patients to be of minimal relevance. This may be due to the different spectrum of symptoms associated with ICI therapy. ICIs induce immune-related adverse events which can affect every organ system compared to, for example, chemotherapy. Most clinical trials used non-specific instruments like the EORTC C30 or FACT-M [16]. The EORTC group is already working on this problem and developing further questionnaires e.g. for immunotherapy. Item libraries exist for common HRQoL instruments, such as the EORTC questionnaires or the PRO-CTCAE, which could help to select suitable instruments for HRQoL assessment in a specific setting [40].

To summarise, the use of a specific instrument for the patient population and the treatment could be useful. A multicentric, longitudinal, standardised study of HRQoL in patients with melanoma during and after ICI therapy is needed to generate a larger dataset to draw confirmatory conclusions. Additionally, a further study for validation of a specific questionnaire for this population is needed.

This study had several limitations. The number of patients included was limited, particularly in the survivor group. This may have resulted in an under- or overrepresentation of certain characteristics. A potential selection bias is possible. Statistical power was reduced, so it is more difficult to recognise the true effect. This also reduces the generalisability of the study. Measuring quality of life is inherently challenging due to the numerous impact factors, that must be considered. The PRO-CTCAE items were specific for the population in our study, but they were not validated for patients with melanoma or patients with ICI therapy. Our item list was conducted by expert opinion including a potential selection bias. HRQoL was assessed at a single point in time and with no baseline data (before treatment) making it difficult to find out whether the reported symptoms were present prior to treatment and maybe attributable to comorbidities. Symptom trajectories or possible persisting irAEs couldn’t be identified as well as causality. This is limiting the interpretation of the symptoms reported by the survivor group. Consequently, the study was unable to draw any confirmatory theses or causality.

The strength of this study is the comparison of HRQoL in patients during and after ICI treatment identifying differences in symptom burden. Additionally, this is a real-world study with inclusion of patients under routine conditions independent of ECOG, comorbidities or other characteristics in contrast to clinical trial participants. Altogether, we could contribute data on symptom burden and HRQoL for patients under and after ICI therapy. Adding an ICI therapy-specific instrument is also a strength of this study.

## Conclusion

The extant data on HRQoL and symptom burden largely confirmed the existing literature. ICI-induced long-term toxicities in melanoma survivors seem to be relevant and need further investigation. Different needs and symptoms in patients on-treatment and off-treatment should be considered what also affects HRQoL measurement.

Rising numbers of patients undergoing ICI therapy and long-term survivors underscore the need for data on HRQoL and symptom burden under routine conditions of these patients. Further research is required to explore additional aspects of the treatment, such as fertility or neurocognition. The development of treatment-specific questionnaires with different attributes for HRQoL measurement should be considered to accurately assess both the total symptom burden and the HRQoL experienced by these patients. A future advanced multicentric, longitudinal study on HRQoL during and after ICI therapy is desirable.

## Supplementary Information

Supplementary Material 1.

Supplementary Material 2.

Supplementary Material 3.

## Data Availability

The datasets used and analysed during the current study are available from the corresponding author on reasonable request.

## Abbreviations

ECOG: Eastern cooperative oncology group
HRQoL: Health-related quality of life
irAE: Immune-related adverse event
ICI: Immune checkpoint inhibitor
PRO-CTCAE: Patient-reported outcomes version of the common terminology criteria for adverse events
EORTC: European organisation for research and treatment of cancer
MID: Minimally important difference
PRO: Patient-reported outcome

## References

[1] Saginala K, Barsouk A, Aluru JS, Rawla P, Barsouk A. Epidemiol Melanoma Med Sci. 2021;9(4):63. 10.3390/medsci9040063. 10.3390/medsci9040063 PMC8544364 34698235

[2] Li Y, Li F, Jiang F, Lv X, Zhang R, Lu A, et al. A Mini-Review for cancer immunotherapy: molecular Understanding of PD-1/PD-L1 pathway & translational Blockade of immune checkpoints. Int J Mol Sci. 2016;17(7):1151. 10.3390/ijms17071151. 27438833 10.3390/ijms17071151 PMC4964524

[3] Haanen JB, Carbonnel F, Robert C, Kerr KM, Peters S, Larkin J, et al. Management of toxicities from immunotherapy: ESMO clinical practice guidelines for diagnosis, treatment and follow-up. Ann Oncol. 2017;28(suppl4):iv119–42. 10.1093/annonc/mdx225. 28881921 10.1093/annonc/mdx225

[4] Wolchok JD, Chiarion-Sileni V, Gonzalez R, Rutkowski P, Grob JJ, Cowey CL, et al. Overall survival with combined Nivolumab and Ipilimumab in advanced melanoma. N Engl J Med. 2017;377(14):1345–56. 10.1056/NEJMoa1709684. 28889792 10.1056/NEJMoa1709684 PMC5706778

[5] Eisemann N, Schumann L, Baltus H, Labohm L, Kraywinkel K, Katalinic A. Longer survival from melanoma in Germany. Dtsch Arztebl Int. 2024;121(2):45–51. 10.3238/arztebl.m2023.0242. 38054977 10.3238/arztebl.m2023.0242 PMC10979441

[6] Langbaum T, Smith TJ. Time to study metastatic-cancer survivorship. N Engl J Med. 2019;380(14):1300–2. 10.1056/NEJMp1901103. 30943335 10.1056/NEJMp1901103

[7] Bloom JR. Surviving and thriving? Psychooncology. 2002;11(2):89–92. 10.1002/pon.606 10.1002/pon.606 11921324

[8] Mayer DK, Nasso SF, Earp JA. Defining cancer survivors, their needs, and perspectives on survivorship health care in the USA. Lancet Oncol. 2017;1e11–8. 10.1016/S1470-2045(16)30573-3. 28049573 10.1016/S1470-2045(16)30573-3

[9] Bullinger M, Quitmann J. Quality of life as patient-reported outcomes: principles of assessment. Dialogues Clin Neurosci. 2014;16(2):137–45. 10.31887/DCNS.2014.16.2/mbullinger. 25152653 10.31887/DCNS.2014.16.2/mbullinger PMC4140508

[10] Bullinger M. In: Kovacs L, Kipke R, Lutz R, editors. Zur messbarkeit von Lebensqualität. Wiesbaden: Springer; 2016.

[11] Valderas JM, Alonso J. Patient reported outcome measures: a model-based classification system for research and clinical practice. Qual Life Res. 2008;17(9):1125–35. 10.1007/s11136-008-9396-4. 18836850 10.1007/s11136-008-9396-4

[12] Wiedemann F, Porzsolt F. Measuring health-related quality of life in randomised controlled trials: expected and reported results do not match. Pragmat Obs Res. 2022;13:9–16. 10.2147/POR.S350165. 35431592 10.2147/POR.S350165 PMC9012498

[13] Petrella TM, Robert C, Richtig E, Miller WH Jr, Masucci GV, Walpole E, et al. Patient-reported outcomes in KEYNOTE-006, a randomised study of pembrolizumab versus ipilimumab in patients with advanced melanoma. Eur J Cancer. 2017;86:115–24. 10.1016/j.ejca.2017.08.032. 28987768 10.1016/j.ejca.2017.08.032

[14] Schadendorf D, Larkin J, Wolchok J, Hodi FS, Chiarion-Sileni V, Gonzalez R, et al. Health-related quality of life results from the phase III checkmate 067 study. Eur J Cancer. 2017;82:80–91. 10.1016/j.ejca.2017.05.031. 28651159 10.1016/j.ejca.2017.05.031 PMC5737813

[15] Schadendorf D, Amonkar MM, Stroyakovskiy D, et al. Health-related quality of life impact in a randomised phase III study of the combination of Dabrafenib and Trametinib versus Dabrafenib monotherapy in patients with BRAF V600 metastatic melanoma. Eur J Cancer. 2015;51(7):833–40. 10.1016/j.ejca.2015.03.004. 25794603 10.1016/j.ejca.2015.03.004

[16] Chen C, Wang Z, Qin YR. Health-related quality of life in stage III-IV melanoma treated with targeted therapy or immunotherapy: a systematic review on the adequacy of reporting and clinical issues in phase III randomized controlled trials. Cancer Med. 2023;12(3):2262–80. 10.1002/cam4.5183. 36030506 10.1002/cam4.5183 PMC9939121

[17] Aaronson NK, Ahmedzai S, Bergman B, Bullinger M, Cull A, Duez NJ, et al. The European organization for research and treatment of cancer QLQ-C30: a quality-of-life instrument for use in international clinical trials in oncology. J Natl Cancer Inst. 1993;85(5):365–76. 10.1093/jnci/85.5.365. 8433390 10.1093/jnci/85.5.365

[18] van Leeuwen M, Kieffer JM, Young TE, Annunziata MA, Arndt V, Arraras JI, et al. Phase III study of the European organisation for research and treatment of cancer quality of life cancer survivorship core questionnaire. J Cancer Surviv. 2023;17(4):1111–30. 10.1007/s11764-021-01160-1. 35088246 10.1007/s11764-021-01160-1

[19] Basch E, Reeve BB, Mitchell SA, Clauser SB, Minasian LM, Dueck AC, et al. Development of the National Cancer Institute’s patient-reported outcomes version of the common terminology criteria for adverse events (PRO-CTCAE). J Natl Cancer Inst. 2014;106(9):dju244. 10.1093/jnci/dju244. 25265940 10.1093/jnci/dju244 PMC4200059

[20] Cocks K, Wells JR, Johnson C, Schmidt H, Koller M, Oerlemans S, et al. Content validity of the EORTC quality of life questionnaire QLQ-C30 for use in cancer. Eur J Cancer. 2023;178:128–38. 10.1016/j.ejca.2022.10.026. 36436330 10.1016/j.ejca.2022.10.026

[21] Fayers PM, Aaronson NK, Bjordal K, Groenvold M, Curran D, Bottomley A, et al. The EORTC scoring manual. 3rd ed. editors. Brussels: European Organisation for Research and Treatment of Cancer; 2001.

[22] Quinten C, Maringwa J, Gotay CC, et al. Patient self-reports of symptoms and clinician ratings as predictors of overall cancer survival. J Natl Cancer Inst. 2011;103(24):1851–8. 10.1093/jnci/djr485. 22157640 10.1093/jnci/djr485 PMC3243678

[23] Nolte S, Waldmann A, Liegl G, Petersen MA, Groenvold M, Rose M. Updated EORTC QLQ-C30 general population norm data for Germany. Eur J Cancer. 2020;137:161–70. 10.1016/j.ejca.2020.06.002. 32777715 10.1016/j.ejca.2020.06.002

[24] Dréno B, Ascierto P, Atkinson V, et al. Health-related quality of life impact of cobimetinib in combination with vemurafenib in patients with advanced or metastatic *BRAF*^V600^ mutation–positive melanoma. Br J Cancer. 2018;118(6):777–84. 10.1038/bjc.2017.488. 29438370 10.1038/bjc.2017.488 PMC5877437

[25] Gogas H, Dummer R, Ascierto PA, et al. Quality of life in patients with BRAF-mutant melanoma receiving the combination encorafenib plus binimetinib: results from a multicentre, open-label, randomised, phase III study (COLUMBUS). Eur J Cancer. 2021;152:116–28. 10.1016/j.ejca.2021.04.028. 34091420 10.1016/j.ejca.2021.04.028

[26] Schadendorf D, Dummer R, Hauschild A, et al. Health-related quality of life in the randomised KEYNOTE-002 study of pembrolizumab versus chemotherapy in patients with ipilimumab-refractory melanoma. Eur J Cancer. 2016;67:46–54. 10.1016/j.ejca.2016.07.018. 27596353 10.1016/j.ejca.2016.07.018

[27] Cheung WY, Bayliss MS, White MK, Stroupe A, Lovley A, King-Kallimanis BL, et al. Humanistic burden of disease for patients with advanced melanoma in Canada. Support Care Cancer. 2018;61985–91. 10.1007/s00520-017-4025-9. 10.1007/s00520-017-4025-9 PMC5919988 29322243

[28] Egeler M, Lai-Kwon J, Tissier R, et al. Real-world health-related quality of life outcomes for patients with resected stage III/IV melanoma treated with adjuvant anti-PD1 therapy. Eur J Cancer. 2024;200:113601. 10.1016/j.ejca.2024.113601. 38340383 10.1016/j.ejca.2024.113601

[29] Warner BM, Baer AN, Lipson EJ, Allen C, Hinrichs C, Rajan A. Et aI. Sicca syndrome associated with immune checkpoint inhibitor therapy. Oncologist. 2019;91259–69. 10.1634/theoncologist.2018-0823. 10.1634/theoncologist.2018-0823 PMC6738284 30996010

[30] Cheung WY, Le LW, Gagliese L, Zimmermann C. Age and gender differences in symptom intensity and symptom clusters among patients with metastatic cancer. Support Care Cancer. 2011;19(3):417–23. 10.1007/s00520-010-0865-2. 20333411 10.1007/s00520-010-0865-2

[31] Walsh D, Donnelly S, Rybicki L. The symptoms of advanced cancer: relationship to age, gender, and performance status in 1,000 patients. Support Care Cancer. 2000;8(3):175–9. 10.1007/s005200050281. 10789956 10.1007/s005200050281

[32] Mamoor M, Postow MA, Lavery JA, Baxi SS, Khan N, Mao JJ, et al. Quality of life in long-term survivors of advanced melanoma treated with checkpoint inhibitors. J Immunother Cancer. 2020;8(1):e000260. 10.1136/jitc-2019-000260. 32152222 10.1136/jitc-2019-000260 PMC7061889

[33] Johnson DB, Nebhan CA, Moslehi JJ, Balko JM. Immune-checkpoint inhibitors: long-term implications of toxicity. Nat Rev Clin Oncol. 2022;19(4):254–67. 10.1038/s41571-022-00600-w. 35082367 10.1038/s41571-022-00600-w PMC8790946

[34] Fischbeck S, Weyer-Elberich V, Zeissig SR, Imruck BH, Blettner M, Binder H, et al. Determinants of illness-specific social support and its relation to distress in long-term melanoma survivors. BMC Public Health. 2018;18(1):511. 10.1186/s12889-018-5401-1. 29665805 10.1186/s12889-018-5401-1 PMC5904995

[35] Rogiers A, Willemot L, McDonald L, et al. Real-world effectiveness, safety, and health-related quality of life in patients receiving adjuvant nivolumab for melanoma in Belgium and Luxembourg: results of preserv mel. Cancers (Basel). 2023;15(19):4823. 10.3390/cancers15194823. 37835517 10.3390/cancers15194823 PMC10572061

[36] Rogiers A, Leys C, Lauwyck J, Schembri A, Awada G, Schwarze JK, et al. Neurocognitive function, psychosocial outcome, and health-related quality of life of the first-generation metastatic melanoma survivors treated with ipilimumab. J Immunol Res. 2020;2020:2192480. 10.1155/2020/2192480. 32775464 10.1155/2020/2192480 PMC7391091

[37] Garutti M, Lambertini M, Puglisi F. Checkpoint inhibitors, fertility, pregnancy, and sexual life: a systematic review. ESMO Open. 2021;6(5):100276. 10.1016/j.esmoop.2021.100276. 34597942 10.1016/j.esmoop.2021.100276 PMC8487000

[38] Salzmann M, Tosev G, Heck M, Schadendorf D, Maatouk I, Enk AH, et al. Male fertility during and after immune checkpoint inhibitor therapy: a cross-sectional pilot study. Eur J Cancer. 2021;152:41–8. 10.1016/j.ejca.2021.04.031. 34062486 10.1016/j.ejca.2021.04.031

[39] Briken P, Matthiesen S, Pietras L, Wiessner C, Klein V, Reed GM, et al. Estimating the prevalence of sexual dysfunction using the new ICD-11 guidelines—results of the first representative, population-based German health and sexuality survey (GeSiD). Dtsch Arztebl Int. 2020;117:653–8. 10.3238/arztebl.2020.0653. 33357346 10.3238/arztebl.2020.0653 PMC7829447

[40] Piccinin C, Basch E, Bhatnagar V, et al. Recommendations on the use of item libraries for patient-reported outcome measurement in oncology trials: findings from an international, multidisciplinary working group. Lancet Oncol. 2023;24(2):e86–95. 10.1016/S1470-2045(22)00654-4. 36725153 10.1016/S1470-2045(22)00654-4

