# Supplementary material for: Health-related quality of life and symptom burden in patients with melanoma during and after immune checkpoint inhibitor therapy – a pilot study
Source: BMC Cancer. 2025 Oct 16;25:1599. doi: 10.1186/s12885-025-15069-w (PMC12532475; doi:10.1186/s12885-025-15069-w)
Supplement: Supplementary file 1 — Supplementary Material 1. [file 12885_2025_15069_MOESM1_ESM.docx]

**STROBE Statement - Checklist of items that should be included in reports of cross-sectional studies**

|  | **Item No.** | **STROBE items** | **Location in manuscript where items are reported** | |
| --- | --- | --- | --- | --- |
| **Title and abstract** | | | |  |
|  | 1 | (a) Indicate the study’s design with a commonly used term in the title or the abstract (b) Provide in the abstract an informative and balanced summary of what was done and what was found | Title page, abstract | |
| **Introduction** | | | |  |
| Background rationale | 2 | Explain the scientific background and rationale for the investigation being reported | Page 5 (Introduction) | |
| Objectives | 3 | State specific objectives, including any prespecified hypotheses | Page 6 (Introduction)  Page 7 (Methods)  Study protocol page 3-4 | |
| **Methods** | | | |  |
| Study Design | 4 | Present key elements of study design early in the paper | Page 6 | |
| Setting | 5 | Describe the setting, locations, and relevant dates, including periods of recruitment, exposure, follow-up, and data collection | Page 6-7  Study protocol page 5 | |
| Participants | 6 | *(a) Cohort study* - Give the eligibility criteria, and the sources and methods of selection of participants. Describe methods of follow-up  *Case-control study* - Give the eligibility criteria, and the sources and methods of case ascertainment and control selection. Give the rationale for the choice of cases and controls  *Cross-sectional study* - Give the eligibility criteria, and the sources and methods of selection of participants  *(b) Cohort study* - For matched studies, give matching criteria and number of exposed and unexposed  *Case-control study* - For matched studies, give matching criteria and the number of controls per case | Page 6-7 (Study population) | |
| Variables | 7 | Clearly define all outcomes, exposures, predictors, potential confounders, and effect modifiers. Give diagnostic criteria, if applicable. | Page 7-10 (outcomes)  Page 20-23 (Discussion – potential confounders) | |
| Data sources/ measurement | 8 | For each variable of interest, give sources of data and details of methods of assessment (measurement).  Describe comparability of assessment methods if there is more than one group | Page 7-9 (Data sources, Measurement) | |
| Bias | 9 | Describe any efforts to address potential sources of bias | Page 7 | |
| Study size | 10 | Explain how the study size was arrived at | Page 7  Stiudy protocol page 7-8 (more detailed) | |
| Quantitative variables | 11 | Explain how quantitative variables were handled in the analyses. If applicable, describe which groupings were chosen, and why | Page 6-7 (treatment and survivor group) | |
| Statistical methods | 12 | (a) Describe all statistical methods, including those used to control for confounding  (b) Describe any methods used to examine subgroups and interactions  (c) Explain how missing data were addressed  (d) *Cohort study* - If applicable, explain how loss to follow-up was addressed  *Case-control study* - If applicable, explain how matching of cases and controls was addressed  *Cross-sectional study* - If applicable, describe analytical methods taking account of sampling strategy  (e) Describe any sensitivity analyses | Page 9-10 (Statistical analyses) | |
| **Results** | | | |  |
| Participants | 13 | (a) Report the numbers of individuals at each stage of the study (*e.g.*, numbers potentially eligible, examined for eligibility, confirmed eligible, included in the study, completing follow-up, and analysed)  (b) Give reasons for non-participation at each stage.  (c) Consider use of a flow diagram | Page 10-11 (Study population), Figure 2 | |
| Descriptive data | 14 | (a) Give characteristics of study participants (*e.g.*, demographic, clinical, social) and information on exposures and potential confounders  (b) Indicate the number of participants with missing data for each variable of interest  (c) *Cohort study* - summarise follow-up time (*e.g.*, average and total amount) | Page 11 (Patient characteristics), Table 1 | |
| Outcome data | 15 | *Cohort study* - Report numbers of outcome events or summary measures over time  *Case-control study* - Report numbers in each exposure category, or summary measures of exposure  *Cross-sectional study* - Report numbers of outcome events or summary measures | Page 13-19 (immune-related events, HRQoL outcomes)  Supplementary tables S1-S2  Table 2, table 4, table 6 | |
| Main results | 16 | (a) Give unadjusted estimates and, if applicable, confounder-adjusted estimates and their precision (e.g., 95% confidence interval). Make clear which confounders were adjusted for and why they were included  (b) Report category boundaries when continuous variables were categorized  (c) If relevant, consider translating estimates of relative risk into absolute risk for a meaningful time period | Page 13-19 (HRQoL outcomes), Table 2-6  Supplementary tables S3-S6 | |
| Other analyses | 17 | Report other analyses done—e.g., analyses of subgroups and interactions, and sensitivity analyses | Page 16 (Subgroup analyses), supplementary tables S7-14 | |
| **Discussion** | | | |  |
| Key results | 18 | Summarise key results with reference to study objectives | Page 20 | |
| Limitations | 19 | Discuss limitations of the study, taking into account sources of potential bias or imprecision. Discuss both direction and magnitude of any potential bias | Page 24 (Discussion) | |
| Interpretation | 20 | Give a cautious overall interpretation of results considering objectives, limitations, multiplicity of analyses, results from similar studies, and other relevant evidence | Page 20-24 (Discussion) | |
| Generalisability | 21 | Discuss the generalisability (external validity) of the study results | Page 23-24 (Discussion) | |
| **Other Information** | | | |  |
| Funding | 22 | Give the source of funding and the role of the funders for the present study and, if applicable, for the original study on which the present article is based | Page 27 | |

Reference: https://www.strobe-statement.org/checklists/
