# Supplementary material for: Health-related quality of life and symptom burden in patients with melanoma during and after immune checkpoint inhibitor therapy – a pilot study
Source: BMC Cancer. 2025 Oct 16;25:1599. doi: 10.1186/s12885-025-15069-w (PMC12532475; doi:10.1186/s12885-025-15069-w)
Supplement: Supplementary file 2 — Supplementary Material 2. [file 12885_2025_15069_MOESM2_ESM.pdf]

Health-related quality of life in patients with malignant melanoma under immune checkpoint inhibitor therapy – comparison of time points during active therapy and one year free of therapy and recurrence:

An observational study

– University Hospital Dresden (UKD) –

automated translation with DeepL

Short title:

Health-related quality of life and symptom burden in patients with melanoma during and after immune checkpoint inhibitor therapy

Acronym: LQ Mel

Study directors Dresden:

Dr. Olaf Schoffer
Prof. Friedegund Meier
Dresden University of Technology
Faculty of Medicine Carl Gustav Carus
Fetscherstr. 74
01307 Dresden

Study coordinators of the overall study:

Dr. Martin Eichler
Dr. Frank Friedrich Gellrich
Lea Pöschmann (doctoral candidate)
Technical University of Dresden
Faculty of Medicine Carl Gustav Carus
Fetscherstr. 74
01307 Dresden

Signatures of the study directors:

Olaf Schoffer   Friedegund Meier

Summary

It will be carried out a comparison of health-related quality of life (HRQoL) in patients with advanced or metastatic malignant melanoma undergoing immune checkpoint inhibitor (ICI) treatment at two time points. Patients undergoing active therapy will be compared with patients who have been free of therapy and recurrence for at least one year. Examining the literature recently made available on HRQoL in patients undergoing ICI treatment reveals that the results of prospective clinical studies are questionable. For example, no reduction in quality of life is apparent in patients receiving combination therapy with nivolumab and ipilimumab with severe side effects in around 60% of patients. Although the currently available patient-reported outcome measures (PROMs) cover important dimensions of health-related quality of life, they may not capture all the experiences and side effects of patients receiving immune checkpoint inhibitor therapy. It would be important to determine whether side effects, symptoms and problems that are particularly relevant for melanoma patients undergoing immune checkpoint inhibitor therapy can be recorded with greater sensitivity using a more specific questionnaire for this patient group.

A total of 140 patients will be surveyed. The data will be evaluated as part of a doctoral thesis.

automated translation with DeepL

1 Introduction/Scientific background

Malignant melanoma is one of the most common types of cancer in Germany (Robert Koch Institute, 2023). Depending on the geographical location in Europe, the incidence is between 3 and 35 cases per 100,000 per year and is rising steadily (Arnold et al., 2022). The main risk factors for developing malignant melanoma are fair skin, UV exposure and genetic predisposition (Itin, 1999). For locally confined melanoma, surgical treatment is the curative procedure of choice. Advanced and metastatic melanoma was primarily treated palliatively with chemotherapy in the past, with a median survival time of six months to one year. In the last decade, a significant improvement in survival has been made thanks to the development of new targeted therapies and immunotherapies. These include, for example, immune checkpoint inhibitors (ICI). Immune checkpoint inhibitors are monoclonal antibodies that inhibit certain checkpoints, such as CTLA-4 or PD-1. This influences the interaction between T lymphocytes and antigen-presenting cells or tumour cells, resulting in increased T cell activity and thus activation of the immune system (Lamos & Hunger, 2020). Immune checkpoint inhibitors are currently approved in stage II, III and IV melanoma (as adjuvant and/or non-adjuvant therapy). In stage IV, nivolumab, pembrolizumab and ipilimumab are used (Zaremba et al., 2020). These substances have significantly improved survival in advanced stages. In the KEYNOTE-006 study, pembrolizumab achieved a 7-year overall survival rate of 38% (Robert et al., 2023). In the CheckMate-067 study, melanoma-specific survival after 7.5 years was 47% for nivolumab and 55% for nivolumab plus ipilimumab (Hodi et al., 2022). However, high toxicity was notable, with 59% of patients experiencing severe side effects. Due to the different mechanism of action of ICI compared to chemotherapy, for example, the side effects differ. These immune-mediated side effects are called as 'immune-related adverse events' (irAEs) and can occur in any organ system. They can be classified into five severity grades by the Common Terminology Criteria for Adverse Events (CTCAE), with hospitalisation indicated

from grade 3 onwards. The time of the onset of side effects is highly variable, ranging from the start of therapy to years after the end of therapy. The most affected organ systems are the skin, gastrointestinal tract and endocrine system (Braun et al., 2020). Due to these complex and sometimes serious side effects, which can be irreversible and in rare cases lethal, health-related quality of life (HRQoL) plays an important role for patients under and after treatment. HRQoL encompasses various dimensions, including physical, social and psychological functions (Ferrans et al., 2005). In the KEYNOTE-006 study, approximately one-fifth of patients receiving pembrolizumab experienced grade 3/4 side effects, most commonly diarrhoea, fatigue and colitis. In the Checkmate 067 study investigating the combination therapy nivolumab-ipilimumab, the percentage was around 59% (Larkin et al., 2015). In the Checkmate 238 study, patients with stage IIIB, IIIC and IV melanoma were treated with ipilimumab or nivolumab. Treatment-related side effects occurred in approximately 85% of patients in the nivolumab group and in over 95% of patients in the ipilimumab group. The most common side effects were fatigue, diarrhoea and pruritus. Nevertheless, no significant difference was found in quality of life, which was assessed using the EORTC QLQ-C30, EQ-5D utility index and EQ-5D VAS (Weber et al., 2017). Given the high percentage of side effects, the lack of reduction in quality of life therefore appears questionable. In large clinical trials of immune checkpoint inhibitors, quality of life was assessed using the EORTC-QLQ-C30 and EQ-5D questionnaires. The evaluation showed no differences compared to the normal population. Non-disease-specific questionnaires, such as the EORTC QLQ-C30, are generally aimed at all oncology patients and cover important dimensions, but do not cover all specific side effects of ICI treatment. However, there is currently no specific quality of life questionnaire for immunotherapy patients. We therefore want to investigate whether a questionnaire from the PRO-CTCAE catalogue that is specifically tailored to this patient group can better capture relevant and typical side effects and symptoms than general instruments such as the EORTC QLQ-C30.

1.1 Objectives of the study (general)

To investigate health-related quality of life and compare the two patient groups with each other and with the results of existing clinical studies. To investigate whether more specific questionnaires can better capture side effects and thus HRQoL than general, less disease-specific questionnaires.

1.2 Target criteria

Primary:

Assessment of the health-related quality of life (HRQoL) of patients during immune checkpoint inhibitor treatment with nivolumab, ipilimumab, pembrolizumab or the combination of nivolumab/ipilimumab using EORTC QLQ-C30 and a self-compiled questionnaire with ICI specific items from the PRO-CTCAE catalogue. This questionnaire is validated and reliable but not for this specific patient population (Hagelstein et al., 2016). The items were selected based on the expert opinion of experienced dermato-oncologists.

automated translation with DeepL

As a comparison, assessment of the HRQoL of patients (survivors) who are free of therapy and recurrence one year after treatment with the above-mentioned immune checkpoint inhibitors.

For this group, use of the EORTC SURV100 questionnaire and the same self-created questionnaire with specific items from the PRO-CTCAE catalogue. Comparison with existing clinical studies and assessment of whether HRQoL can be measured more accurately using more specific items.

The construct and content validity are important for evaluating this comparison. Content validity describes the extent to which the test instrument completely captures the characteristic being investigated. In this regard, the EORTC and the self-developed questionnaire should be compared. The aim is to determine whether a relevant proportion of symptoms that is higher than the proportion of randomly occurring symptoms in the overall population is captured.

2 Study-related measures / study procedure

Participants in the study will be asked to complete two questionnaires:

- 1a for the group undergoing active treatment (treatment group): EORTC QLQ-C30
- 1b for the recurrence-free group at least one year after the end of treatment (survivor group): EORTC SURV100
- for both groups: specific questionnaire from the PRO-CTCAE catalogue

The first 10 participants in the survivor group will be interviewed face-to-face about the SURV100 questionnaire, as phase IV of testing is currently beginning for the German version.

In addition, the questionnaires will be sent by post to those in the recurrence-free group who do not have a follow-up appointment at the UKD.

2.1 General study procedure

A total of 140 patients are to be recruited, all of whom will be included by the UKD.

Potential study participants will be identified using the calendar function in “Orbis” (hospital information system) and approached during follow-up examinations or therapy.

It is assumed that not all of those approached will participate in the survey, so we are assuming about 150 patient contacts. A declaration of consent (Appendix 3) and the study questionnaires (Appendices 5-7) will be handed out. If the person agrees to participate in the study, they sign the declaration of consent and complete the questionnaire.

In addition, the questionnaires will be sent by post to those in the recurrence-free group who do not have a follow-up appointment at the UKD. If Orbis has consent to contact patients for research purposes, they will be contacted directly. If consent to oncological research has not been given, patients will be informed about the research project by telephone or in writing

automated translation with DeepL

and asked if they are willing to participate. The questionnaires will only be sent out once participation has been confirmed.

2.2 Detailed study procedure

2.2.1 Identification and recruitment of potential study participants

After contacting patients during follow-up appointments or appointments to receive immunotherapy, they will be informed and receive a consent form and the two questionnaires. It takes approximately 40 minutes to complete the questionnaires.

Patients contacted by post receive a letter (Appendix 4), patient information (Appendices 2a and 2b) and a consent form. Patients who have previously agreed to be contacted again will receive the questionnaires in this letter. All other patients will receive the questionnaires only after consent to participation in the study has been obtained. If the person agrees to participate in the study, they return the signed consent form and the completed questionnaire to the Dresden study centre using the enclosed postage-paid envelopes.

2.2.2 Review of the consent form, storage of study documents, data comparison

If the consent form is incomplete and therefore not legally valid, the study centre will contact the study participant again and ask them to complete the consent form. If a complete and legally valid declaration of consent is received, it will be archived for 10 years in an access-protected area.

2.2.3 Transmission of questionnaire data and clinical data

If the participant has agreed to participate in the study, all data will be stored on a protected server at the University Hospital Dresden.

The original questionnaire will remain in a locked room at the UKD until the end of the study in case any questions arise.

The stored paper questionnaires will be destroyed by 31 December 2024 at the latest. If no consent to data transfer or study participation has been given, the questionnaires will not be scanned and will be destroyed immediately.

2.2.4 Expected start and end of the study

- Start of recruitment (first patient in): 1 May 2023
- End of recruitment (last patient out): 31 December 2023
- Evaluation and final report: July 2023 – December 2024

2.3 Data protection concept

automated translation with DeepL

At all stages of the study project and at all participating institutions, personal data and health-related study data are strictly separated. All data and the key table remain at the study centre in Dresden. Only those involved in the study have access to the data, and access to the key table is restricted to the doctoral candidate. The key table is located on a protected server at the University Hospital Dresden and will be destroyed after the data collection has been completed, at the latest by 31 December 2024. After that, it will no longer be possible to link the study data to personally identifiable data, and the data will be anonymised.

2.4 Burden on participants

Completion of two questionnaires (time required: 40 minutes).

2.5 Benefits and risks

- Study participants do not derive any personal benefit from participating in the study.
- Being reminded of cancer and its associated consequences may have psychological consequences.
- Participation is voluntary and can be discontinued at any time.

2.6 Study type/study design

Cross-sectional study (no intervention, only survey)

2.7 Inclusion criteria

- Minimum age: 18 years (at diagnosis)
- Advanced or metastatic malignant melanoma, diagnosis must be histologically confirmed
- Treatment with a PD1 immune checkpoint inhibitor (nivolumab, pembrolizumab) or combination therapy of nivolumab and the CTLA-4 inhibitor ipilimumab
- Able to read and answer questionnaires
- Able to give informed consent

2.8 Exclusion criteria

- Any psychiatric illness or cognitive impairment that would prevent completion of the questionnaires.
- Insufficient knowledge of the German language
- No signed declaration of consent
- Patients with a too severe illness in the opinion of the recruiting physician

automated translation with DeepL

2.9 Randomisation procedure/plan

Not applicable.

2.10 Discontinuation criteria

Individual discontinuation criteria: see 2.8

3 Statistical design

3.1 Statistical methods

All statistical tests are two-sided and differences are considered significant if $p<0.05$. Missing data in the C30 questionnaire are handled according to the EORTC QLG guidelines.

The data from patients who assess HRQoL problems and the completed questionnaires are analysed using basic quantitative analyses, including descriptive statistics (e.g. % missing data, mean values and standard deviations, floor and ceiling effects, etc.). Responses regarding the relevance of the topics will be analysed based on priority ratings (number of patients who rated each item as a priority) and the range of responses for each item. The responses to the EORTC QLQ-C30 are analysed based on prevalence (number of patients who experienced the respective complaint, i.e. who gave 2, 3 or 4 points, divided by the total number of patients who completed the relevant item, multiplied by 100) and the range of responses for each item. If necessary, a factor analysis will be performed.

3.2 Number of cases

A total of approximately 140 individuals were surveyed for this study.

The results of the KEYNOTE-054 study (pembrolizumab vs. placebo) were used for the analysis of the number of cases. A total of 1,019 patients were included in the study. Quality of life was assessed using the General Health Questionnaire in the form of the EORTC QLQ-C30.

The 514 patients in the pembrolizumab group had a mean GHQ score of 77.55 points with a standard deviation of 18.2. In the placebo group with 505 patients, the mean score was 76.54 points with a standard deviation of 17.81. During therapy, there was a difference of -1.1 points between the groups. After therapy, the difference was -2.2 points. Over a two-year period, the difference between the two groups was -2.2 points (Bottomley et al., 2021). Overall, the variation in QoL scores is very high. An effect of 2.2 points difference in the score corresponds to an effect size of approximately 0.11 with these standard deviations. With a significance level of 5% and a power of 80%, this difference would only be detectable in 1300 + 1300 patients, i.e. a total of 2600 patients. A study of this size would only be possible as a multicentre study and should only be conducted with sufficient prior knowledge of whether the data can be transferred to the melanoma patients considered here. The KEYNOTE-054 study only included patients with resected high-risk stage III melanoma without prior systemic

automated translation with DeepL

therapy and an ECOG of 0 or 1. Further exclusion criteria were autoimmune diseases, uncontrolled infections and systemic therapy with glucocorticoids. We would like to include all patients in routine care in our study. This includes patients with stage II, III and IV melanoma, regardless of prior treatment, comorbidities and ECOG. Therefore, the present study is being conducted as a pilot study to determine whether the values for the quality of life scores, their variation and group differences (here treatment and survivor groups) are confirmed. In addition, the pilot study will examine whether more specific items, as opposed to general questionnaires such as the QLQ-C30, potentially provide greater differentiation between the groups. This will be done by including all eligible patients who agree to participate over a period of 8 months. The number of cases that can be achieved in the pilot study does not allow for confirmatory conclusions, which is why it is exploratory in nature.

4 Legal and ethical aspects

4.1 Declaration of Helsinki

The study will be conducted in accordance with the Declaration of Helsinki in its current version.

4.2 Ethics committee

The study will be submitted to the responsible ethics committee at the Technical University of Dresden and the documents to be used will be submitted for review prior to the start of the study.

4.3 Voluntary participation

Participation by patients is voluntary.

4.4 Withdrawal

Consent may be withdrawn by the patient at any time without giving reasons and without any disadvantages for further medical care.

4.5 Data deletion upon withdrawal

Upon withdrawal from the study, any data already collected will be destroyed.

However, this is only possible as long as the assignment of the study ID to the name has not yet been deleted.

After that, the data will be completely anonymised and it will no longer be possible to determine which questionnaire belongs to a specific person.

automated translation with DeepL

4.6 Information and consent

Study participants will be informed verbally and in writing about the nature and scope of the planned study before the study begins. Their consent will be documented by their signature on the consent form.

4.7 Approval according to X-ray regulation/radiation protection regulation

Not applicable

4.8 Funding, sponsors, institutional affiliations, possible conflicts of interest, incentives for test subjects, compensation

There is no funding or other financial support.

5 References

Arnold, M., Singh, D., Laversanne, M., Vignat, J., Vaccarella, S., Meheus, F., Cust, A. E., de Vries, E., Whiteman, D. C., & Bray, F. (2022). Global Burden of Cutaneous Melanoma in 2020 and Projections to 2040. *Journal of the American Medical Association Dermatology*, *158*(5), 495-503. https://doi.org/10.1001/jamadermatol.2022.0160

Bottomley, A., Coens, C., Mierzynska, J., Blank, C. U., Mandalà, M., Long, G. V., . . . Group, E. M. (2021). Adjuvant pembrolizumab versus placebo in resected stage III melanoma (EORTC 1325-MG/KEYNOTE-054): health-related quality-of-life results from a double-blind, randomised, controlled, phase 3 trial. *Lancet Oncol*, *22*(5), 655-664. https://doi.org/10.1016/S1470-2045(21)00081-4

Braun, G. S., Kirschner, M., Rübben, A., Wahl, R. U., Amann, K., Benesova, K., & Leipe, J. (2020). [Side effects of novel cancer immunotherapies]. *Nephrologe*, *15*(3), 191-204. https://doi.org/10.1007/s11560-020-00424-8

Ferrans, C. E., Zerwic, J. J., Wilbur, J. E., & Larson, J. L. (2005). Conceptual Model of Health-Related Quality of Life. *Journal of Nursing Scholarship*, *37*(4), 336-342. https://doi.org/https://doi.org/10.1111/j.1547-5069.2005.00058.x

Hagelstein, V., Ortland, I., Wilmer, A., Mitchell, S. A., & Jaehde, U. (2016). Validation of the German patient-reported outcomes version of the common terminology criteria for adverse events (PRO-CTCAE™). *Ann Oncol*, *27*(12), 2294-2299. https://doi.org/10.1093/annonc/mdw422

Hodi, F. S., -Sileni, V. C., Lewis, K. D., Grob, J.-J., Rutkowski, P., Lao, C. D., . . . Larkin, J. (2022). Long-term survival in advanced melanoma for patients treated with nivolumab plus ipilimumab in CheckMate 067. *Journal of Clinical Oncology*, *40*(16_suppl), 9522-9522. https://doi.org/10.1200/JCO.2022.40.16_suppl.9522

Itin, P. H. (1999). [Risk factors for the development of malignant melanomas]. *Ther Umsch*, *56*(6), 298-301. https://doi.org/10.1024/0040-5930.56.6.298

Lamos, C., & Hunger, R. E. (2020). [Checkpoint inhibitors-indications and application in melanoma patients]. *Z Rheumatol*, *79*(8), 818-825. https://doi.org/10.1007/s00393-020-00870-8

Larkin, J., Chiarion-Sileni, V., Gonzalez, R., Grob, J. J., Cowey, C. L., Lao, C. D., . . . Wolchok, J. D. (2015). Combined Nivolumab and Ipilimumab or Monotherapy in Untreated Melanoma. *N Engl J Med*, *373*(1), 23-34. https://doi.org/10.1056/NEJMoa1504030

automated translation with DeepL

Robert, C., Carlino, M.S., McNeil, C., Ribas, A., Grob, J.-J., Schachter, J., . . . Long, G. V. (2023). Seven-year follow-up of the phase III KEYNOTE-006 study: Pembrolizumab versus ipilimumab in advanced melanoma. *Journal of Clinical Oncology*, 41, 3998–4003. https://doi.org/10.1200/JCO.22.01599

Robert Koch Institute. (2023). Cancer in Germany for 2019/2020. 14th edition. In Robert Koch-Institute (Ed.) and Gesellschaft der epidemiologischen Krebsregister in Deutschland e.V. (Ed.). Berlin.

Weber, J., Mandala, M., Del Vecchio, M., Gogas, H. J., Arance, A. M., Cowey, C. L., . . . Collaborators, C. (2017). Adjuvant Nivolumab versus Ipilimumab in Resected Stage III or IV Melanoma. *N Engl J Med*, *377*(19), 1824-1835. https://doi.org/10.1056/NEJMoa1709030

Zaremba, A., Zimmer, L., Griewank, K. G., Ugurel, S., Roesch, A., Schadendorf, D., & Livingstone, E. (2020). Immuntherapie beim malignen Melanom. *Der Internist*, *61*(7), 669-675. https://doi.org/10.1007/s00108-020-00812-1

automated translation with DeepL
