# Supplementary material for: Health-related quality of life and symptom burden in patients with melanoma during and after immune checkpoint inhibitor therapy – a pilot study
Source: BMC Cancer. 2025 Oct 16;25:1599. doi: 10.1186/s12885-025-15069-w (PMC12532475; doi:10.1186/s12885-025-15069-w)
Supplement: Supplementary file 3 — Supplementary Material 3. [file 12885_2025_15069_MOESM3_ESM.docx]

**Supplementary tables:**

**Table S1: Treatment-related adverse events in the treatment group**

|  | **Nivolumab**  **(n=40)** | | **Pembrolizumab**  **(n=17)** | **Nivolumab/**  **Ipilimumab**  **(n=16)** | | **ICI (all) (n=73)** |
| --- | --- | --- | --- | --- | --- | --- |
| **Severity level CTCAE** | 1-4 | 3-4 | 1-4 | 1-4 | 3-4 | 1-4 |
| Colitis | 3 (7.5%) | 0 | 1 (5.9%) | 4 (25.0%) | 4 (25.0%) | 8 (11.0%) |
| Pneumonitis | 2 (5.0%) | 0 | 0 | 0 | 0 | 2 (2.7%) |
| Hepatitis | 3 (7.5%) | 2 (5.0%) | 0 | 3 (18.8%) | 3 (18.8%) | 6 (8.2%) |
| Fatigue | 3 (7.5%) | 0 | 1 (5.9%) | 0 | 0 | 4 (5.5%) |
| Hypophysitis | 0 | 0 | 0 | 1 (6.3%) | 0 | 1 (1.4%) |
| Thyreoditis | 3 (7.5%) | 0 | 0 | 0 | 0 | 3 (4.1%) |
| Gastritis | 1 (2.5%) | 0 | 0 | 0 | 0 | 1 (1.4%) |
| Pancreatitis | 2 (5.0%) | 0 | 0 | 0 | 0 | 2 (2.7%) |
| Xerostomia | 1 (2.5%) | 0 | 0 | 0 | 0 | 1 (1.4%) |
| Neurological side effects | 3 (7.5%) | 1 (2.5%) | 2 (11.8%) | 0 | 0 | 5 (6.8%) |
| Pruritus | 0 | 0 | 1 (5.9%) | 0 | 0 | 1 (1.4%) |
| Arthritis | 4 (10%) | 0 | 2 (11.8%) | 0 | 0 | 6 (8.2%) |
| Exanthema | 0 | 0 | 0 | 2 (12.5%) | 1 (6.3%) | 2 (2.7%) |
| Nephritis | 2 (5.0%) | 1 (2.5%) | 0 | 0 | 0 | 2 (2.7%) |
| Myocarditis | 0 | 0 | 0 | 1 (6.3%) | 0 | 1 (1.4%) |
| Thrombosis | 1 (2.5%) | 0 | 0 | 0 | 0 | 1 (1.4%) |
| Meningitis | 0 | 0 | 0 | 1 (6.3%) | 1 (6.3%) | 1 (1.4%) |
| **Number of patients** | **13 (32.5%)** | **5 (7.5%)** | **7 (41.2%)** | **11 (68.7%)** | **7 (43,8%)** | **31 (42,5%)** |

*Collected via electronic health records. Severity of adverse events classified according to Common Criteria of Adverse Events (CTCAE) version 5.0 by the National Cancer Institute, grades 3-4 classified as severe events, n number of patients*

**Table S2: Treatment-related adverse events in the survivor group**

|  | **Nivolumab**  **(n=13)** | **Pembrolizumab**  **(n=12)** | **Nivolumab/**  **Ipilimumab**  **(n=1)** | **ICI (all) (n=26)** |
| --- | --- | --- | --- | --- |
| Colitis | 1 (7.7%) | 4 (33.3%) | 0 | 5 (19.2%) |
| Pneumonitis | 0 | 1 (8.3%) | 0 | 1 (3.8%) |
| Hepatitis | 1 (7.7%) | 0 | 0 | 1 (3.8%) |
| Fatigue | 1 (7.7%) | 1 (8.3%) | 0 | 2 (7.7%) |
| Hypophysitis | 0 | 1 (8.3%) | 0 | 1 (3.8%) |
| Thyreoditis | 1 (7.7%) | 1 (8.3%) | 0 | 2 (7.7%) |
| Pruritus | 0 | 1 (8.3%) | 0 | 1 (3.8%) |
| Vitiligo | 1 (7.7%) | 0 | 0 | 1 (3.8%) |
| Neurological side effects | 0 | 1 (8.3%) | 1 (100) | 2 (7.7%) |
| Arthritis | 2 (15.4%) | 3 (25.0%) | 0 | 5 (19.2%) |
| Nephritis | 1 (7.7%) | 0 | 0 | 1 (3.8%) |
| **Number of patients** | **5 (38.5%)** | **6 (50.0%)** | **1 (100)** | **12 (46.2%)** |

*Only ICI-related toxicities. Includes all toxicities occurring during and after ICI therapy. Collected via electronic health records.*

*N number of patients, CTCAE classification not collected*

**Table S3: Results of the PRO-CTCAE in the treatment group**

| **Symptom** | **n** | **Absolute frequency** | **Relative frequency (%)** |
| --- | --- | --- | --- |
| Xerosis cutis | 73 | 49 | 67.1 |
| Pruritus | 73 | 37 | 50.7 |
| Xerostomia | 73 | 36 | 49.3 |
| Problems erection | 23 | 11 | 47.8 |
| Reduced sexual interest | 36 | 17 | 47.2 |
| Aching joints | 73 | 33 | 45.2 |
| Memory loss | 73 | 31 | 42.5 |
| Hyp-/dysesthesia | 73 | 30 | 41.1 |
| Edema | 73 | 29 | 39.7 |
| Appetite loss | 73 | 29 | 39.7 |
| Aching muscles | 73 | 24 | 32.9 |
| Cardiac arrythmia | 73 | 24 | 32.9 |
| Dizziness | 73 | 24 | 32.9 |
| Cough | 73 | 23 | 31.5 |
| Problems ejaculation | 23 | 7 | 30.4 |
| Blurred vision | 73 | 22 | 28.8 |
| Headache | 73 | 21 | 26.0 |
| Hot flash | 73 | 18 | 24.7 |
| Abdominal pain | 73 | 18 | 24.7 |
| Diarrhoea | 73 | 16 | 21.9 |
| Exanthema | 73 | 15 | 20.5 |
| Further Symptom | 73 | 13 | 17.8 |
| Nausea | 73 | 12 | 16.4 |
| Chills | 73 | 8 | 11.0 |
| Vomiting | 73 | 4 | 5.5 |

*N number pf patients*

**Table S4: PRO-CTCAE items with frequency often/almost constantly**

|  | **Treatment group** | | | **Survivor group** | | |
| --- | --- | --- | --- | --- | --- | --- |
| **Symptom** | **n** | **Absolut frequency** | **Relative frequency** | **n** | **Absolut frequency** | **Relative frequency** |
| Edema | 73 | 12 | 16.4 | 26 | 4 | 15.4 |
| Aching joints | 73 | 12 | 16.4 | 26 | 6 | 23.1 |
| Problems ejaculation | 23 | 2 | 11.5 | 12 | 1 | 8.3 |
| Aching muscles | 73 | 6 | 8.2 | 26 | 1 | 3.8 |
| Hot flash | 73 | 5 | 6.8 | 26 | 0 | 0.0 |
| Chill | 73 | 4 | 5.5 | 26 | 1 | 3.8 |
| Headache | 73 | 4 | 5.5 | 26 | 0 | 0.0 |
| Cardiac arrhythmia | 73 | 4 | 5.5 | 26 | 2 | 7.7 |
| Diarrhea | 73 | 4 | 5.5 | 26 | 1 | 3.8 |
| Nausea | 73 | 3 | 4.1 | 26 | 0 | 0.0 |
| Abdominal pain | 73 | 2 | 2.7 | 26 | 0 | 0.0 |
| Vomiting | 73 | 1 | 1.4 | 26 | 0 | 0.0 |

*Table includes PRO-CTCAE items with frequency CTCAE grade 3-4*

*n= number of responses*

**Table S5: PRO-CTCAE items with strength severe/very severe**

|  | **Treatment group** | | | | **Survivor group** | | |
| --- | --- | --- | --- | --- | --- | --- | --- |
| **Symptom** | **n** | | **Absolut frequency** | **Relative frequency** | **n** | **Absolut frequency** | **Relative frequency** |
| Xerosis cutis | 73 | 18 | | 24.7 | 26 | 4 | 15.4 |
| Pruritus | 73 | 16 | | 21.9 | 26 | 2 | 7.7 |
| Aching joints | 73 | 11 | | 15.1 | 26 | 5 | 19.2 |
| Xerostomia | 73 | 10 | | 13.7 | 26 | 2 | 7.7 |
| Edema | 73 | 9 | | 12.3 | 26 | 1 | 3.8 |
| Hyp-/Dysesthesia | 73 | 9 | | 12.3 | 26 | 3 | 11.5 |
| Reduced sexual interest | 36 | 4 | | 11.1 | 14 | 0 | 0.0 |
| Appetite loss | 73 | 8 | | 11.0 | 26 | 0 | 0.0 |
| Aching muscles | 73 | 6 | | 8.2 | 26 | 1 | 3.8 |
| Hot flash | 73 | 5 | | 6.8 | 26 | 0 | 0.0 |
| Dizziness | 73 | 5 | | 6.8 | 26 | 1 | 3.8 |
| Chill | 73 | 4 | | 5.5 | 26 | 1 | 0.0 |
| Blurred vision | 73 | 4 | | 5.5 | 26 | 3 | 11.5 |
| Problems erection | 23 | 1 | | 4.3 | 12 | 2 | 16.7 |
| Memory loss | 73 | 3 | | 4.1 | 26 | 2 | 7.7 |
| Cardiac arrhythmia | 73 | 3 | | 4.1 | 26 | 1 | 3.8 |
| Abdominal pain | 73 | 3 | | 4.1 | 26 | 0 | 0.0 |
| Nausea | 73 | 3 | | 4.1 | 26 | 0 | 0.0 |
| Headache | 73 | 2 | | 2.7 | 26 | 0 | 0.0 |
| Cough | 73 | 1 | | 1.4 | 26 | 3 | 11.5 |
| Vomiting | 73 | 1 | | 1.4 | 26 | 0 | 0.0 |

*Table includes PRO-CTCAE items with strength CTCAE grade 3-4*

*N=number of responses*

**Table S6:** **PRO-CTCAE items with interference strong/very strong**

|  | **Treatment group (n=73)** | | **Survivor group (n=26)** | |
| --- | --- | --- | --- | --- |
| **Symptom** | **Absolut frequency** | **Relative frequency** | **Absolut frequency** | **Relative frequency** |
| Edema | 8 | 11.0 | 0 | 0.0 |
| Hyp-/Dysesthesia | 8 | 11.0 | 1 | 3.8 |
| Dizziness | 8 | 11.0 | 1 | 3.8 |
| Aching joints | 7 | 9.6 | 2 | 7.7 |
| Memory loss | 5 | 6.8 | 2 | 7.7 |
| Aching muscles | 5 | 6.8 | 1 | 3.8 |
| Blurred vision | 4 | 5.5 | 1 | 3.8 |
| Headache | 3 | 4.1 | 0 | 0.0 |
| Appetite loss | 3 | 4.1 | 0 | 0.0 |
| Cough | 3 | 4.1 | 2 | 7.7 |
| Abdominal pain | 3 | 4.1 | 0 | 0.0 |

*Table includes PRO-CTCAE items with interference CTCAE grade 3-4*

*N=number of responses*

**Table S7:** EORTC QLQ-C30 analysed by gender

| **Scale** | **male (n=40)** | **female (n=33)** | **p-value** | **MID** |
| --- | --- | --- | --- | --- |
| Global Health Score | 63.5 | 57.8 | **<0.001 *** |  |
| Physical function | 78.5 | 74.1 | **<0.001 *** |  |
| Role function | 73.3 | 72.2 | **<0.001 *** |  |
| Emotional function | 73.8 | 62.4 | **<0.001 *** | * |
| Cognitive function | 86.3 | 83.8 | **<0.001 *** |  |
| Social function | 76.7 | 74.7 | **<0.001 *** |  |
| Fatigue | 37.8 | 41.4 | **<0.001 *** |  |
| Nausea | 4.2 | 9.6 | 0.053 |  |
| Pain | 22.9 | 26.8 | **0.005 *** |  |
| Dyspnoea | 19.2 | 19.2 | 0.092 |  |
| Insomnia | 25.8 | 36.4 | **<0.001 *** | * |
| Appetite loss | 16.7 | 22.2 | 0.219 |  |
| Constipation | 5.8 | 12.1 | 0.053 |  |
| Diarrhoea | 7.5 | 15.2 | 0.095 |  |
| Financial difficulties | 11.7 | 8.1 | 0.157 |  |

*A p-value of <0.05 was considered as significant, * means p<0.05*

*MID=minimally important difference, analogue to clinical trials a difference of ≥10 points was considered as relevant, * means difference ≥10 points*

**Table S8:** EORTC QLQ-C30 analysed by immune checkpoint inhibitor

| **Scale** | **Ipilimumab/ Nivolumab (n=16)** | **Nivolumab (n=40)** | **Pembrolizumab (n=17)** | **p- value** | **MID** |
| --- | --- | --- | --- | --- | --- |
| Global Health Score | 52.6 | 62.3 | 65.7 | 0.343 | * |
| Physical function | 76.7 | 76.8 | 75.7 | 0.713 |  |
| Role function | 76.0 | 72.5 | 70.6 | 0.941 |  |
| Emotional function | 60.4 | 67.1 | 79.9 | **0.042 *** | * |
| Cognitive function | 86.5 | 83.8 | 87.3 | 0.876 |  |
| Social function | 81.3 | 73.3 | 76.5 | 0.439 |  |
| Fatigue | 47.9 | 38.9 | 32.7 | 0.298 | * |
| Nausea | 15.6 | 4.6 | 2.9 | 0.308 | * |
| Pain | 21.9 | 26.3 | 23.5 | 0.751 |  |
| Dyspnoea | 16.7 | 20.8 | 17.7 | 0.909 |  |
| Insomnia | 27.1 | 35.0 | 23.5 | 0.489 | * |
| Appetite loss | 31.2 | 15.0 | 17.7 | 0.399 | * |
| Constipation | 12.5 | 6.7 | 9.8 | 0.929 |  |
| Diarrhoea | 25.0 | 6.7 | 7.8 | **0.043 *** | * |
| Financial difficulties | 4.2 | 15.0 | 3.9 | 0.100 | * |

*A p-value of <0.05 was considered as significant, * means p<0.05*

*MID=minimally important difference, analogue to clinical trials a difference of ≥10 points was considered as relevant, * means difference ≥10 points*

**Table S9:** EORTC QLQ-C30 analysed by stage (AJCC version 8)

| **Scale** | **Stage II**  **(n=7)** | **Stage III (n=16)** | **Stage IV (n=47)** | **p- value** | **MID** |
| --- | --- | --- | --- | --- | --- |
| Global Health Score | 76.2 | 56.3 | 61.2 | 0.128 | * |
| Physical function | 86.7 | 79.6 | 73.9 | 0.722 | * |
| Role function | 95.2 | 76.0 | 67.7 | 0.062 | * |
| Emotional function | 84.5 | 69.3 | 66.0 | 0.174 | * |
| Cognitive function | 95.2 | 85.4 | 83.3 | 0.472 | * |
| Social function | 88.1 | 80.2 | 72.3 | 0.412 | * |
| Fatigue | 17.5 | 43.8 | 41.6 | 0.137 | * |
| Nausea | 0 | 7.3 | 7.1 | 0.348 |  |
| Pain | 11.9 | 17.7 | 30.5 | 0.069 | * |
| Dyspnoea | 14.3 | 16.7 | 22.0 | 0.493 |  |
| Insomnia | 14.3 | 27.1 | 34.0 | 0.528 | * |
| Appetite loss | 4.8 | 33.3 | 16.3 | 0.107 | * |
| Constipation | 9.5 | 2.1 | 11.4 | 0.242 |  |
| Diarrhoea | 0 | 16.7 | 11.4 | 0.301 | * |
| Financial difficulties | 4.8 | 18.8 | 8.5 | 0.292 | * |

*A p-value of <0.05 was considered as significant, * means p<0.05*

*MID=minimally important difference, analogue to clinical trials a difference of ≥10 points was considered as relevant, * means difference ≥10 points*

*Stages were defined according to the Cancer Staging Manual of the American Joint Committee on Cancer (AJCC) 8^th^ edition*

**Table S10:** EORTC QLQ-C30 analysed by age group

| **Scale** | **<50 years (n=9)** | **50-59 years (n=13)** | **60-69 years (n=15)** | **>70 years (n=36)** | **p- value** | **MID** |
| --- | --- | --- | --- | --- | --- | --- |
| Global Health Score | 77.8 | 67.9 | 63.3 | 53.2 | **0.004 *** | * |
| Physical function | 85.9 | 82.6 | 77.3 | 71.7 | **0.042 *** | * |
| Role function | 81.5 | 71.2 | 76.7 | 69.4 | 0.533 | * |
| Emotional function | 69.4 | 64.1 | 70.0 | 69.4 | 0.762 |  |
| Cognitive function | 79.6 | 83.3 | 85.6 | 87.0 | 0.557 |  |
| Social function | 74.1 | 70.5 | 74.4 | 78.7 | 0.563 |  |
| Fatigue | 30.9 | 39.3 | 34.8 | 43.5 | 0.442 | * |
| Nausea | 7.4 | 3.8 | 3.3 | 8.8 | 0.761 |  |
| Pain | 29.6 | 23.1 | 21.1 | 25.5 | 0.867 |  |
| Dyspnoea | 7.4 | 18.0 | 13.3 | 25.0 | 0.222 | * |
| Insomnia | 18.5 | 30.8 | 33.3 | 32.4 | 0.727 | * |
| Appetite loss | 7.4 | 10.3 | 13.3 | 27.8 | 0.126 | * |
| Constipation | 14.8 | 5.1 | 8.9 | 8.3 | 0.949 |  |
| Diarrhea | 3.7 | 10.3 | 2.2 | 16.7 | 0.248 | * |
| Financial difficulties | 7.4 | 30.8 | 4.4 | 5.6 | **<0.001*** | * |

*A p-value of <0.05 was considered as significant, * means p<0.05*

*MID=minimally important difference, analogue to clinical trials a difference of ≥10 points was considered as relevant, * means difference ≥10 points*

**Table S11:** EORTC QLQ-C30 analysed by performance status (ECOG)

| **Scale** | **ECOG 0 (n=37)** | **ECOG 1 (n=26)** | **ECOG 2-3 (n=10)** | **p- value** | **MID** |
| --- | --- | --- | --- | --- | --- |
| Global Health Score | 68.2 | 56.1 | 46.7 | **0.002 *** | * |
| Physical function | 87.4 | 70.3 | 52.7 | **<0.001*** | * |
| Role function | 82.4 | 67.3 | 51.7 | **0.007 *** | * |
| Emotional function | 69.6 | 69.9 | 61.7 | 0.453 |  |
| Cognitive function | 86.9 | 84.0 | 81.7 | 0.583 |  |
| Social function | 74.3 | 77.6 | 76.7 | 0.855 |  |
| Fatigue | 29.4 | 44.4 | 63.3 | **0.002 *** | * |
| Nausea | 6.8 | 3.8 | 13.3 | 0.219 |  |
| Pain | 21.2 | 26.3 | 33.3 | 0.793 | * |
| Dyspnoea | 9.9 | 30.8 | 23.3 | **0.008 *** | * |
| Insomnia | 22.5 | 41.0 | 33.3 | 0.116 | * |
| Appetite loss | 12.6 | 23.1 | 33.3 | 0.057 | * |
| Constipation | 6.3 | 11.5 | 10.0 | 0.159 |  |
| Diarrhoea | 10.8 | 10.3 | 13.3 | 0.989 |  |
| Financial difficulties | 12.6 | 10.3 | 0.0 | 0.156 | * |

*A p-value of <0.05 was considered as significant, * means p<0.05*

*MID=minimally important difference, analogue to clinical trials a difference of ≥10 points was considered as relevant, * means difference ≥10 points*

*Performance status graded by Eastern Cooperative Oncology Group (ECOG) performance status scale*

**Table S12:** EORTC QLQ-C30 analysed by previous therapies

| **Scale** | **No previous therapy (n=30)** | **previous therapy (n=43)** | **p- value** | **MID** |
| --- | --- | --- | --- | --- |
| Global Health Score | 57.5 | 63.4 | **<0.001*** |  |
| Physical function | 73.8 | 78.4 | **<0.001*** |  |
| Role function | 75.0 | 71.3 | **<0.001*** |  |
| Emotional function | 70.3 | 67.4 | **<0.001*** |  |
| Cognitive function | 82.8 | 86.8 | **<0.001*** |  |
| Social function | 76.1 | 75.6 | **<0.001*** |  |
| Fatigue | 40.0 | 39.0 | **<0.001*** |  |
| Nausea | 2.8 | 9.3 | **0.002 *** |  |
| Pain | 18.9 | 28.7 | 0.054 |  |
| Dyspnoea | 24.4 | 15.5 | 0.470 |  |
| Insomnia | 32.2 | 29.5 | **0.008 *** |  |
| Appetite loss | 18.9 | 19.4 | 0,817 |  |
| Constipation | 8.9 | 8.5 | **0.002 *** |  |
| Diarrhoea | 5.6 | 14.7 | **0.004 *** |  |
| Financial difficulties | 12.2 | 8.5 | **0.009 *** |  |

*A p-value of <0.05 was considered as significant, * means p<0.05*

*MID=minimally important difference, analogue to clinical trials a difference of ≥10 points was considered as relevant, * means difference ≥10 points*

*Previous therapy includes other prior systemic treatment, intralesional therapy or radiation*

**Table S13:** EORTC QLQ-C30 analysed by occurrence of irAEs

| **Scale** | **No irAE (n=42)** | **irAE (n=31)** | **p- value** | **MID** |
| --- | --- | --- | --- | --- |
| Global Health Score | 65.9 | 54.3 | **<0.001*** | * |
| Physical function | 75.2 | 78.3 | **<0.001*** |  |
| Role function | 73.8 | 71.5 | **<0.001*** |  |
| Emotional function | 74.8 | 60.2 | **<0.001*** | * |
| Cognitive function | 86.1 | 83.9 | **<0.001*** |  |
| Social function | 79.4 | 71.0 | **<0.001*** |  |
| Fatigue | 36.2 | 43.7 | **<0.001*** |  |
| Nausea | 2.8 | 11.8 | 0.093 |  |
| Pain | 27.4 | 21.0 | **0.003 *** |  |
| Dyspnoea | 16.7 | 22.6 | 0.059 |  |
| Insomnia | 32.5 | 28.0 | **<0.001*** |  |
| Appetite loss | 10.3 | 31.2 | 0.150 | * |
| Constipation | 7.9 | 9.7 | 0.093 |  |
| Diarrhoea | 7.1 | 16.1 | 0.155 |  |
| Financial difficulties | 11.1 | 8.6 | 0.244 |  |

*A p-value of <0.05 was considered as significant, * means p<0.05*

*MID=minimally important difference, analogue to clinical trials a difference of ≥10 points was considered as relevant, * means difference ≥10 points*

*irAE=immune-related adverse event, related to ICI therapy*

**Table S14:** EORTC QLQ-C30 analysed by occurrence of brain metastases

| **Scale** | **No brain metastases (n=56)** | **Brain metastases (n=17)** | **p- value** | **MID** |
| --- | --- | --- | --- | --- |
| Global Health Score | 59.1 | 67.2 | **<0.001*** |  |
| Physical function | 76.1 | 78.0 | **<0.001*** |  |
| Role function | 72.9 | 72.5 | **<0.001*** |  |
| Emotional function | 69.2 | ^[[1]](#footnote-1)^66.7 | **<0.001*** |  |
| Cognitive function | 86.9 | 79.4 | **<0.001*** |  |
| Social function | 76.5 | 73.5 | **<0.001*** |  |
| Fatigue | 38.3 | 43.1 | **<0.001*** |  |
| Nausea | 4.8 | 12.7 | 0.770 |  |
| Pain | 22.0 | 33.3 | **<0.001*** | ***** |
| Dyspnoea | 21.4 | 11.8 | **<0.001*** |  |
| Insomnia | 29.2 | 35.3 | **<0.001*** |  |
| Appetite loss | 20.2 | 15.7 | **0.002 *** |  |
| Constipation | 7.7 | 11.8 | 0.770 |  |
| Diarrhoea | 8.9 | 17.6 | 0.596 |  |
| Financial difficulties | 8.9 | 13.7 | 0.446 |  |

*A p-value of <0.05 was considered as significant, * means p<0.05*

*MID=minimally important difference, analogue to clinical trials a difference of ≥10 points was considered as relevant, * means difference ≥10 points*

*Occurrence of brain metastases refers to time of the survey*

**Table S15: Results of the EORTC SURV100**

| **scale** | **n** | **mean** | **SD** | **range** |
| --- | --- | --- | --- | --- |
| **Functional scales** | | | | |
| Global Health Score | 26 | 67.0 | 19.4 | 25.0-100 |
| Physical function | 26 | 69.0 | 26.8 | 0-100 |
| Cognitive function | 26 | 81.7 | 20.6 | 41.7-100 |
| Emotional function | 26 | 79.1 | 20.1 | 33.3-100 |
| Role function | 26 | 71.8 | 28.8 | 0-100 |
| Body image | 26 | 83.3 | 23.6 | 16.7-100 |
| Symptom awareness | 26 | 19.2 | 25.3 | 0-100 |
| Positive health behavior change | 26 | 34.6 | 34.0 | 0-100 |
| Positive life outlook | 26 | 44.9 | 31.1 | 0-100 |
| Positive impact on behaviour others | 26 | 31.4 | 30.3 | 0-100 |
| Positive social functioning | 26 | 41.0 | 34.4 | 0-100 |
| Sexual functioning | 19 | 78.3 | 31.1 | 0-100 |
| Work | 9 | 87.0 | 17.7 | 58.3-100 |
| Sex enjoyable | 14 | 83.3 | 28.5 | 0-100 |
| Relationship with partner stronger | 21 | 73.0 | 37.4 | 0-100 |
| **Symptom scale** | | | | |
| Fatigue | 26 | 34.3 | 26.9 | 0-83.3 |
| Sleep | 26 | 34.6 | 26.9 | 0-91.7 |
| Pain | 26 | 25.6 | 21.7 | 0-83.3 |
| Social interference | 26 | 20.5 | 19.6 | 0-66.7 |
| Health distress | 26 | 52.1 | 32.2 | 0-100 |
| Negative health outlook | 26 | 38.6 | 26.3 | 4.8-90.5 |
| Social isolation | 26 | 31.4 | 33.8 | 0-100 |
| Sexual problems when active | 14 | 20.2 | 23.7 | 0-66.7 |
| Sexual problems | 19 | 21.7 | 31.1 | 0-100 |
| **Single Items** |  |  |  |  |
| Financial difficulties | 26 | 11.5 | 23.0 | 0-100 |
| Risk of family members getting cancer | 26 | 44.9 | 38.8 | 0-100 |
| Deeper meaning to cancer | 26 | 50.0 | 44.5 | 0-100 |
| Treated differently | 26 | 12.8 | 16.5 | 0-33.3 |
| Problems insurance, loans, mortgage | 11 | 6.1 | 20.1 | 0-66.7 |
| Lost income | 13 | 25.6 | 30.9 | 0-100 |
| Concerned about having children | 5 | 6.7 | 14.9 | 0-33.3 |
| Worry impact cancer on child | 22 | 68.2 | 33.3 | 0-100 |
| Dry vagina | 3 | 66.7 | 57.7 | 0-100 |
| Problems erection | 15 | 37.8 | 43.4 | 0-100 |
| **Symptom checklist** |  |  |  |  |
| Dyspnea | 26 | 20.5 | 28.4 | 0-100 |
| Aching joints | 26 | 39.7 | 26.7 | 0-66.7 |
| Aching muscles | 26 | 24.4 | 25.9 | 0-66.7 |
| Sensitive to cold or heat | 26 | 23.1 | 30.9 | 0-100 |
| Cold acra | 26 | 25.6 | 33.1 | 0-100 |
| Dysesthesia of hands | 26 | 19.2 | 25.3 | 0-66.7 |
| Dysesthesia of feet | 26 | 28.2 | 38.5 | 0-100 |
| Skin problems | 26 | 33.3 | 36.5 | 0-100 |
| Restless legs | 26 | 19.2 | 32.9 | 0-100 |
| Difficulties for long standing | 26 | 39.7 | 36.5 | 0-100 |
| Edema | 26 | 23.1 | 29.5 | 0-100 |
| Muscle cramps | 26 | 10.3 | 18.3 | 0-66.7 |
| Muscle weakness | 26 | 11.5 | 24.7 | 0-100 |
| Being cold fast | 26 | 24.4 | 32.1 | 0-100 |
| pyrosis | 26 | 12.8 | 21.2 | 0-66.7 |
| Feeling sick | 26 | 19.2 | 25.3 | 0-66.7 |
| Problems with weight | 26 | 19.2 | 23.4 | 0-100 |

*N number of responses, SD standard deviation*

**Table S16: Results of QLQ-C30 for both groups**

| **Scale** | **Treatment group** | **Survivor group** | **p-value** | **MID** | **German population norm data** |
| --- | --- | --- | --- | --- | --- |
| Global Health Score | 61.0 (±21.6) | 67.0 (±19.3) | 0.207 |  | 67.0 (±21.8) |
| Physical function | 76.5 (±22.8) | 76.7 (±24.5) | 0.898 |  | 82.8 (±21.2) |
| Role function | 72.8 (±28.9) | 74.4 (±28.8) | 0.804 |  | 80.8 (±27.2) |
| Emotional function | 68.6 (±22.8) | 74.0 (±23.5) | 0.311 |  | 73.9 (±24.7) |
| Cognitive function | 85.2 (±20.8) | 82.1 (±20.5) | 0.393 |  | 83.9 (±22.7) |
| Social function | 75.8 (±26.2) | 79.5 (±19.6) | 0.780 |  | 84.8 (±25.5) |
| Fatigue | 39.4 (±27.8) | 35.5 (±28.1) | 0.550 |  | 31.5 (±27.2) |
| Nausea | 6.6 (±16.8) | 1.4 (±4.1) | 0.203 |  | 6.0 (±17.2) |
| Pain | 24.6 (±30.2) | 25.6 (±21.7) | 0.423 |  | 27.6 (±30.9) |
| Dyspnoea | 19.2 (±27.2) | 20.5 (±28.4) | 0.857 |  | 18.7 (±27.3) |
| Insomnia | 30.6 (±35.0) | 35.9 (±37.6) | 0.539 |  | 27.6 (±33.1) |
| Appetite loss | 19.2 (±29.4) | 6.7 (±15.1) | **0.042 *** | ***** | 10.1 (±23.3) |
| Constipation | 8.7 (±19.3) | Not applicable | - | - | 9.6 (±22.3) |
| Diarrhoea | 10.0 (±24.3) | 12.5 (±21.5) | 0.488 |  | 10.4 (±22.7) |
| Financial difficulties | 10.0 (±20.6) | 11.5 (±23.0) | 0.750 |  | 11.3 (±25.0) |

*For the survivor group scales were calculated with the help of SURV100.*

*A p-value of <0,05 was considered as significant.*

*MID=minimally important difference analogue to clinical trials, * means difference ≥10 points*

*German population norm data by Nolte et al. 2020 (weighted by sex and age)*

**Table S17: Results of PRO-CTCAE for both groups**

|  | **Treatment group** | | | **Survivor group** | | |
| --- | --- | --- | --- | --- | --- | --- |
| **Symptom** | **n** | **Absolut frequency** | **relative frequency** | **n** | **Absolut frequency** | **relative frequency** |
| Xerosis cutis | 73 | 49 | 67.1 | 26 | 18 | 69.2 |
| Pruritus | 73 | 37 | 50.7 | 26 | 12 | 46.2 |
| Xerostomia | 73 | 36 | 49.3 | 26 | 9 | 34.6 |
| Problems erection | 23 | 11 | 47.8 | 12 | 4 | 33.3 |
| Reduced sexual interest | 36 | 17 | 47.2 | 14 | 7 | 50.0 |
| Aching joints | 73 | 33 | 45.2 | 26 | 20 | 76.9 |
| Memory loss | 73 | 31 | 42.5 | 26 | 14 | 53.8 |
| Hyp-/Dysesthesia | 73 | 30 | 41.1 | 26 | 14 | 53.8 |
| Edema | 73 | 29 | 39.7 | 26 | 8 | 30.8 |
| Appetite loss | 73 | 29 | 39.7 | 26 | 5 | 19.2 |
| Aching muscles | 73 | 24 | 32.9 | 26 | 7 | 26.9 |
| Cardiac arrhythmia | 73 | 24 | 32.9 | 26 | 11 | 42.3 |
| Dizziness | 73 | 24 | 32.9 | 26 | 7 | 26.9 |
| Cough | 73 | 23 | 31.5 | 26 | 10 | 38.5 |
| Problems ejaculation | 23 | 7 | 30.4 | 12 | 4 | 33.3 |
| Blurred vision | 73 | 22 | 28.8 | 26 | 5 | 19.2 |
| Headache | 73 | 21 | 26.0 | 26 | 6 | 23.1 |
| Hot flash | 73 | 18 | 24.7 | 26 | 11 | 42.3 |
| Abdominal pain | 73 | 18 | 24.7 | 26 | 7 | 26.9 |
| Diarrhoea | 73 | 16 | 21.9 | 26 | 8 | 30.8 |
| Exanthema | 73 | 15 | 20.5 | 26 | 4 | 15.4 |
| Other symptom | 73 | 13 | 17.8 | 26 | 6 | 23.1 |
| Nausea | 73 | 12 | 16.4 | 26 | 3 | 11.5 |
| Chill | 73 | 8 | 11.0 | 26 | 4 | 15.4 |
| Vomiting | 73 | 4 | 5.5 | 26 | 0 | 0.0 |

*N number of patients*

1. [↑](#footnote-ref-1)
